# Immune-responsive gene 1/itaconate activates nuclear factor erythroid 2-related factor 2 in microglia to protect against spinal cord injury in mice

**DOI:** 10.1038/s41419-022-04592-4

**Published:** 2022-02-10

**Authors:** Libin Ni, Jian Xiao, Di Zhang, Zhenxuan Shao, Chongan Huang, Sheng Wang, Yaosen Wu, Naifeng Tian, Liaojun Sun, Aimin Wu, Yifei Zhou, Xiangyang Wang, Xiaolei Zhang

**Affiliations:** 1 Department of Orthopaedics, The Second Affiliated Hospital and Yuying Children’s Hospital of Wenzhou Medical University, Wenzhou, Zhejiang Province China; 2 Key Laboratory of Orthopaedics of Zhejiang Province, Wenzhou, Zhejiang Province China; 3 The Second School of Medicine, Wenzhou Medical University, Wenzhou, Zhejiang Province China; 4 Department of Orthopedic Surgery, The Second Affiliated Hospital, Zhejiang University School of Medicine, Hangzhou, Zhejiang Province China; 5 Chinese Orthopaedic Regenerative Medicine Society, Hangzhou, Zhejiang Province China

**Keywords:** Apoptosis, Cell death in the nervous system, Microglia, Trauma

## Abstract

The pathophysiology of spinal cord injury (SCI) involves primary injury and secondary injury. Secondary injury is a major target for SCI therapy, whereas microglia play an important role in secondary injury. The immunoresponsive gene 1 (Irg-1) has been recorded as one of the most significantly upregulated genes in SCI tissues in gene chip data; however, its role in SCI remains unclear. This study aims to illustrate the role of Irg-1 as well as its regulated metabolite itaconate in SCI. It was demonstrated that the expression of Irg-1 was increased in spinal cord tissues in mice as well as in microglia stimulated by lipopolysaccharides (LPS). It was also shown that overexpression of Irg-1 may suppress LPS-induced inflammation in microglia, while these protective effects were attenuated by Nrf2 silencing. In vivo, overexpression of Irg-1 was shown to suppress neuroinflammation and improve motor function recovery. Furthermore, treatment of microglia with itaconate demonstrated similar inflammation suppressive effects as Irg-1 overexpression in vitro and improved motor function recovery in vivo. In conclusion, the current study shows that Irg-1 and itaconate are involved in the recovery process of SCI, either Irg-1 overexpression or itaconate treatment may provide a promising strategy for the treatment of SCI.

## Introduction

Traumatic spinal cord injury is a critical medical condition with serious consequences and one of the leading causes of disability and death worldwide [1, 2]. Traumatic spinal cord injury causes primary and secondary neurological damage [3]. Primary injury is spinal cord structural injury caused by trauma [4]. The secondary damage is long-lasting, and its pathological process includes oxidative stress, mitochondrial dysfunction, inflammation, apoptosis, etc. [2, 5, 6]. During this process, microglia, which are the innate immune cells of the central nervous system (CNS), secrete inflammation-related toxins and cytokines in response to mechanical injury, further aggravating nerve cell injury [7]. In addition, the interaction between activated microglia and injured neurons is the basis of the pathological and repair processes of the injured CNS [8]. Although there are no satisfactory methods and strategies for the treatment of SCI in clinical trials, targeting inflammation caused by activation of microglia has been considered as a potential treatment for spinal cord injury [9].

The immunoresponsive gene 1 (Irg-1), also known as Acod1, was first identified as a 2.3-kb cDNA from a murine macrophage cell line stimulated with lipopolysaccharide (LPS) [10]. The gene expression profiles in mouse macrophages and microglia have shown that Irg-1 is one of the most significantly increased genes under proinflammatory conditions, such as bacterial infection [11–13]. Irg-1 plays an important role in metabolism and immunity, and individuals with low Irg-1 expression show higher levels of inflammation, indicating that the regulation of Irg-1 expression might be a potential treatment for spinal cord injury [14–16].

In addition, Irg-1 is a mitochondrial-related enzyme that regulates metabolism and participates in the synthesis of a metabolite called itaconate during inflammation [16, 17]. Itaconate levels can reach 5 mM in bone marrow-derived macrophages of mice after LPS stimulation and a large number of studies have shown that itaconate can regulate metabolism and suppress inflammation through various mechanisms, such as by activating the nuclear factor erythroid 2-related factor 2 (Nrf2) pathway [15, 18–20]. Although the Irg-1/itaconate axis plays an important role in metabolism, immunity, and the regulation of inflammation in macrophages, the role of Irg-1 in spinal cord injury and the therapeutic effect of itaconate on spinal cord injury are not known.

In this study, the expression of Irg-1 increased in mice with spinal cord injury and microglia stimulated by LPS. After Irg-1 was knocked down, the level of inflammation increased, but the level of inflammation in Irg-1 overexpression group decreased, and Irg-1 played this role by activating the Nrf2 pathway. In vivo, Irg-1 also showed a promotion effect on the functional recovery of mice with spinal cord injury. In addition, itaconate, a metabolite regulated by Irg-1, also showed an excellent anti-inflammatory effect. Our data demonstrated that itaconate can activate the Nrf2 pathway, inhibit M1 microglia polarization, promote M2 microglia polarization and inhibit LPS-induced inflammation, thus protecting the vitality of nerve cells. Altogether, our study showed that Irg-1/itaconate/Nrf2 axis could alleviate the inflammatory reaction of microglia, and targeting Irg-1 and applying itaconate might be a potential treatment for spinal cord injury.

## Results

### Irg-1 is upregulated in mouse spinal cord injury tissues and LPS-stimulated microglia

**Fig. 1:**
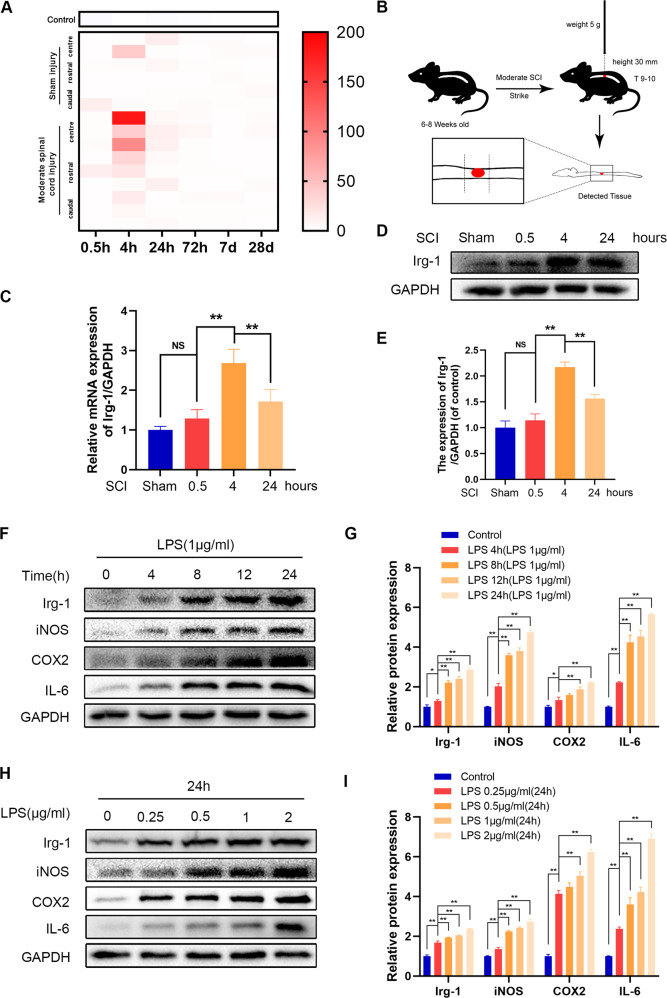
Irg-1 expression in mice with spinal cord injury and LPS-stimulated microglia. **A** The data from the GEO DataSets (https://www.ncbi.nlm.nih.gov/gds), series accession: GDS2159/1427381. **B** Modeling mode of moderate spinal cord injury in mice. **C** The level of Irg-1 mRNA at 0.5, 4, and 24 h after spinal cord injury in mice were detected by qRT-PCR. **D, E** The Irg-1 protein levels at 0.5, 4, and 24 h after spinal cord injury in mice were detected by western blot and quantified by Image J software. **F**–**I** The inflammation level and Irg-1 protein expression of microglia under different time and concentration of LPS stimulation were detected by western blot. The data are presented as the mean ± SD. *n* = 5. NS not significant, *P < 0.05, **P < 0.01.

First, we screened data from the GEO DataSets (https://www.ncbi.nlm.nih.gov/gds) and found from the GDS2159/1427381 dataset that shortly after spinal cord injury in mice, the content of Irg-1 gene at the injured site increased significantly (Fig. 1A). To further determine the changes in the expression of Irg-1 in the acute phase of spinal cord injury in mice, we collected the spinal cord of mice with spinal cord injury at different time points and extracted RNA and protein. A schematic diagram of the construction of the spinal cord injury model is shown in Fig. 1B. The results showed that the expression of Irg-1 mRNA and protein increased in the spinal cord injury model and reached the highest value 4 h after injury, and slightly decreased after 24 h (Fig. 1C–E). LPS was used to stimulate microglia and establish a time and concentration gradient in vitro. The results showed that with the increase in time and the concentration of LPS, the expression of inflammation-related proteins such as iNOS, COX2, and IL-6 increased, and simultaneously, the expression of Irg-1 increased (Fig. 1F–I). These results indicated that Irg-1 is abnormally expressed in spinal cord injury models in vitro and in vivo, and may play a role in the progression of spinal cord injury.

### Irg-1 suppresses inflammation in microglia

**Fig. 2:**
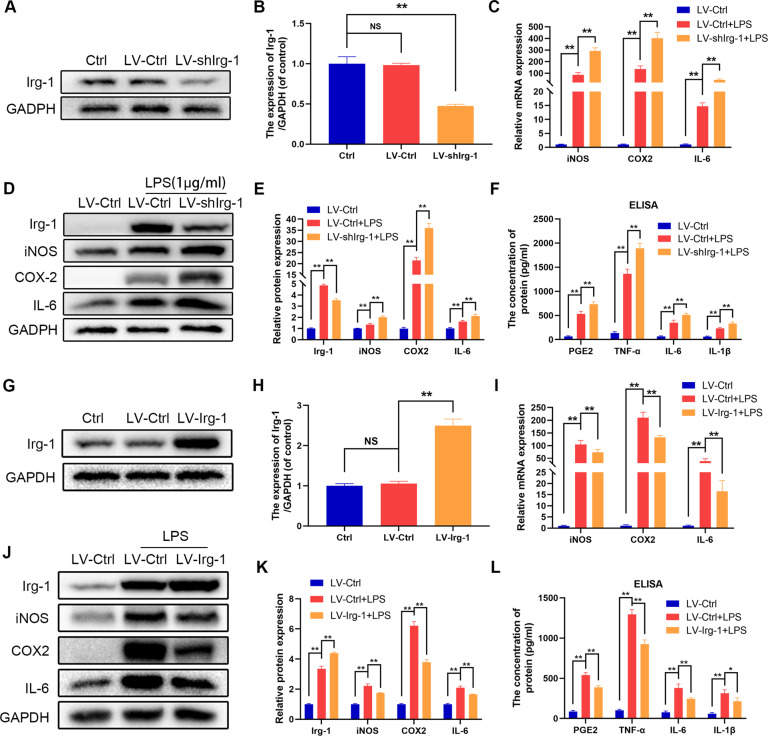
The effect of knockdown and overexpression of Irg-1 on microglia stimulated by LPS. **A**, **B**, **G**, **H** The efficiency of Irg-1 knockdown or overexpression was tested by western blot and quantified by Image J software. **C**, **I** The mRNA expression of iNOS, COX2 and IL-6 were measured by qRT-PCR in the microglia, treated with LV-shIrg-1 or LV-Irg-1. **D**, **E**, **J**, **K** The protein expression of Irg-1, iNOS, COX2 and IL-6 were detected by western blot. **F**, **L** The levels of PGE2, TNF-α, IL-6, IL-1β were detected by ELISA. The data are presented as the mean ± SD. *n* = 5. NS not significant, *P < 0.05, **P < 0.01.

Since Irg-1 level was elevated in microglia stimulated by LPS and spinal cord injury in mice, we studied the relationship between the expression of Irg-1 and inflammation with the increase in the intensity of LPS stimulation. Lentivirus was used to knockdown Irg-1, and the efficiency of lentivirus is shown in Fig. 2A, B. After stimulating microglia with LPS, the results of western blot analysis and quantitative real-time polymerase chain reaction (qRT-PCR) showed that the expression of iNOS, COX2, and IL-6 was higher in microglia with lentiviral knockdown Irg-1 (Fig. 2C–E). The results of ELISA showed that microglia with knocked down Irg-1 produced more inflammatory mediators, such as PGE2, TNF-α, IL-1β, and IL-6 (Fig. 2F). However, when Irg-1 was overexpressed by lentivirus and treated under the same conditions, the result was opposite (Fig. 2G–L), which indicated that Irg-1 plays a protective role in LPS-induced inflammation of microglia.

### Nrf2 mediates the protective effect of Irg-1 against LPS-induced inflammation

**Fig. 3:**
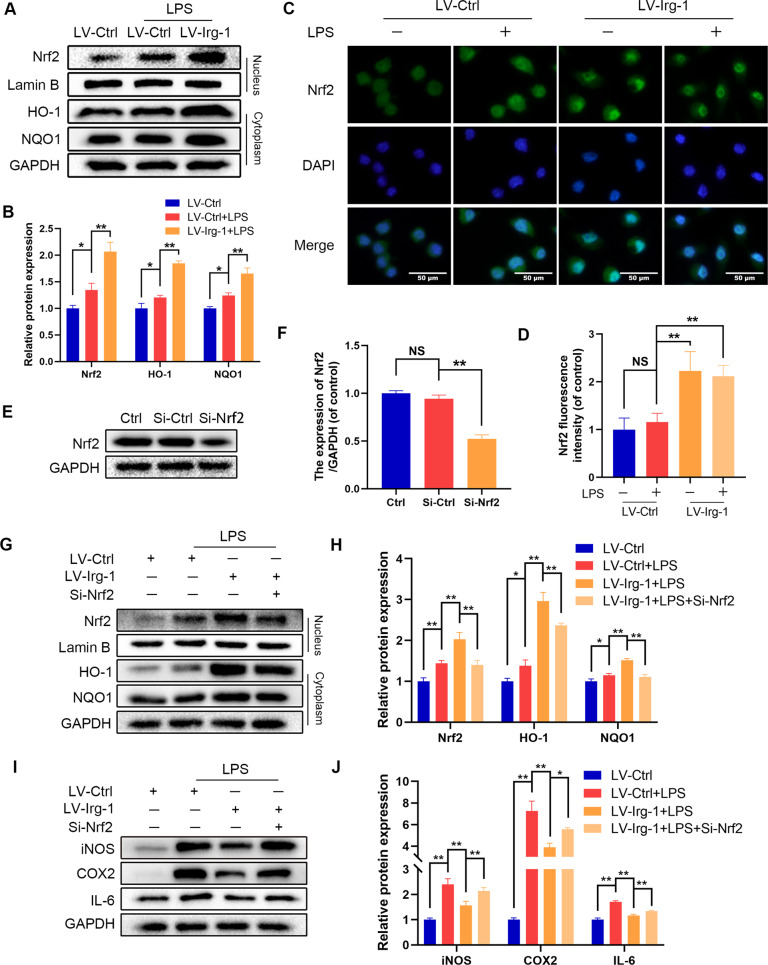
Overexpression of Irg-1 activates the Nrf2 pathway. **A**, **B** The translocation of Nrf2 into the nucleus and cytoplasm of HO-1 and NQO1 expression were detected by western blot and quantified by Image J software. **C**, **D** The Nrf2 was detected by immunofluorescence combined with DAPI staining for nuclei (scale bar: 50 μm). **E**, **F** The silencing efficiency of Si-Nrf2 was detected by western blot. **G**, **H** After treatment with Si-Nrf2, Nrf2 in the nucleus and HO-1 and NQO1 in the cytoplasm were detected with western blot and quantified by Image J software. **I**, **J** The protein expression of iNOS, COX2, IL-6 were detected by western blot. The data are presented as the mean ± SD. *n* = 5. NS not significant, *P < 0.05, **P < 0.01.

After discovering the protective effect of Irg-1 on LPS-induced inflammation in microglia, we investigated its mechanism. As shown in Fig. 3A, B, after overexpression of Irg-1, the expression of Nrf2 in the nucleus increased, and the expression of Nrf2-ARE-dependent proteins HO-1 and NQO1 increased, suggesting that the Nrf2 pathway was activated after the overexpression of Irg-1. In addition, the results of immunofluorescence also showed that after overexpression of Irg-1, the translocation of Nrf2 into the nucleus increased (Fig. 3C, D and Supplementary Fig. S3A, B). Then RNA interference against Nrf2 was performed to evaluate its role in the protection of Irg-1 against LPS-induced inflammation (Fig. 3E, F and Supplementary Fig. S3C, D). The results of the western blot analysis showed that the level of iNOS, COX2 and IL-6 was higher with Nrf2 siRNA transfection compared to their level in the microglia with overexpressed Irg-1 (Fig. 3G–J). These results indicated that Irg-1 can suppress inflammation through the Nrf2 pathway.

### Overexpression of Irg-1 promotes spinal cord injury recovery in mice

**Fig. 4:**
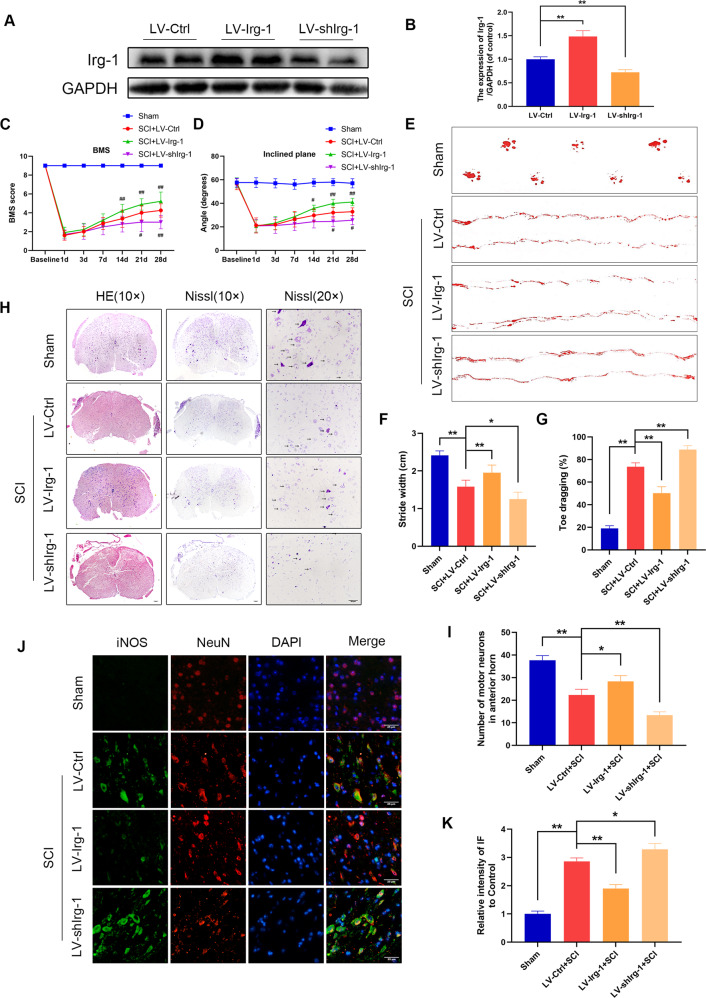
Effect of Irg-1 on inflammation and functional recovery in mice with spinal cord injury. **A**, **B** The effect of lentivirus on spinal cord injury in mice was detected at day 14 by western blot and quantified by Image J software. **C**, **D** Basso mouse scale (BMS) and inclined plane test for the indicated groups at days 1, 3, 7, 14, 21, and 28 (*n* = 8–12). ^#^*P* < 0.05, ^##^*P* < 0.01 relative to the SCI+LV-Ctrl group. **E** Photos of mice footprints at day 28 after SCI. **F**, **G** Quantification of stride width and toe dragging in the footprint analysis. **H** The transected spinal cord sections from each group were analyzed at day 28 by HE staining and Nissl staining. The black arrows indicate motor neurons (scale bar: 100 μm and 50 μm). **I** Quantitative analysis of Nissl positive motor neurons in the anterior horn of spinal cord in each group. **J**, **K** The iNOS/NeuN in spinal cord injury was detected at day 28 by immunofluorescence combined with DAPI staining for nuclei (scale bar: 25 μm). The data are presented as the mean ± SD. *n* = 6 (except for BMS and inclined plane test). *P < 0.05, **P < 0.01.

To evaluate the role of Irg-1 in spinal cord injury, we injected lentivirus into the spinal cord tissue of mice to knockdown or overexpress Irg-1 and evaluate its effect on the progression of spinal cord injury. The efficiency of lentivirus was detected by western blot analysis (Fig. 4A, B).

In spinal cord injury, there is a close relationship between functional recovery and neuron survival. We evaluated behavioral changes using basso mouse scale (BMS) scores, inclined plane test and footprint analysis. The BMS scores and the results of the inclined plane test showed SCI mice with LV-shIrg-1 displayed a lower functional recovery rate and lower maximum scores than SCI mice, but SCI mice in which Irg-1 was overexpressed, recovered relatively well (Fig. 4C, D). Also, differences in the tracks of the posterior limbs were evaluated by footprint analysis (Fig. 4E–G). The footprint of the SCI group was discontinuous, and the distance between the posterior limbs was narrower than the Sham group, which indicated that the posterior limb movement of the SCI mice is severely impaired. This was alleviated in LV-Irg-1 treated mice, while the LV-shIrg-1 group was the most severely affected. Moreover, hematoxylin and eosin (H&E) staining and Nissl staining were performed to observe the morphology of the spinal cord and the neurons. As shown in Fig. 4H, I, the number of neurons in the SCI group was significantly reduced compared with the mice in the Sham group; however, LV-Irg-1 treatment improved this condition. In addition, immunofluorescence results showed that the expression level of iNOS was higher in SCI mice with Irg-1 knockdown (Fig. 4J, K). These results indicated that Irg-1 played a protective role in spinal cord injury of mice.

### Itaconate inhibits inflammation in LPS-treated microglia

**Fig. 5:**
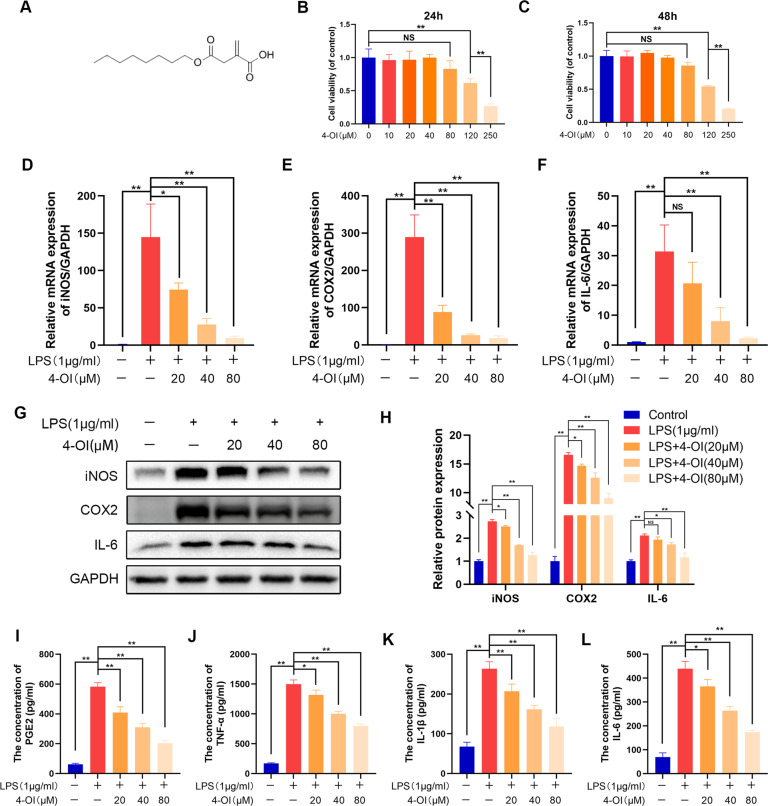
4-OI reduces LPS-induced inflammation in microglia. **A** Chemical structure of 4-OI. **B**, **C** Cell Counting Kit-8 (CCK-8) results for microglia treated with different concentrations of 4-OI for 24 and 48 h. **D**–**F** The mRNA expression of iNOS, COX2 and IL-6 were measured by qRT-PCR. **G**, **H** The protein expression of iNOS, COX2 and IL-6 were measured by western blot and quantified by Image J software. **I**–**L** The expression of PGE2, TNF-α, IL-1β and IL-6 in microglia were measured through ELISA assay. The data are presented as the mean ± SD. *n* = 5. NS not significant, *P < 0.05, **P < 0.01.

To determine whether itaconate, as a metabolite regulated by Irg-1, has the same protective effect as overexpression of Irg-1, we used a cell-permeable itaconate derivative, 4-octyl itaconate (4-OI); its molecular structure is shown in Fig. 5A [19]. First, we used a CCK-8 kit to detect the toxicity of 4-OI to microglia. The concentration of 4-OI at 120 μM showed toxic effects on microglia after 24 h (Fig. 5B, C). Next, we evaluated the effect of 4-OI on the LPS-induced inflammation model. The changes in the protein expression levels of iNOS, COX2 and IL-6 were detected by western blot analysis, and changes in mRNA levels were detected by qRT-PCR. After LPS treatment, the mRNA and protein expression levels of iNOS, COX2 and IL-6 were significantly upregulated (Fig. 5D–H). After 4-OI was added, the upward trend was reversed in a dose-dependent manner. In addition, the mediators of inflammation were measured by ELISA kits. We found that the level of expression of PGE2, TNF-α, IL-1β and IL-6 in the itaconate treatment group was significantly decreased compared to that in the LPS stimulation group (Fig. 5I–L). These results indicated that itaconate can counteract microglia inflammation induced by LPS.

### Itaconate promotes M2 polarization in microglia

**Fig. 6:**
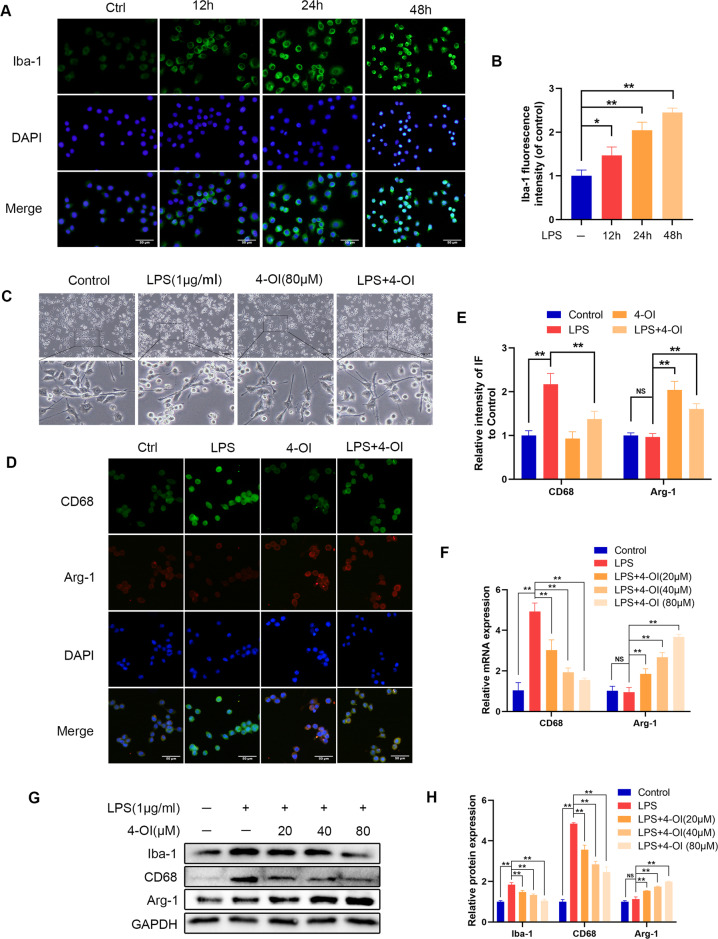
Effect of 4-OI on the polarization of microglia. **A**, **B** The Iba-1 was detected by immunofluorescence combined with DAPI staining for nuclei (scale bar: 50 μm). **C** The morphology of microglia after adding 4-OI (scale bar: 100 μm). **D**, **E** The CD68/Arg-1 was detected by immunofluorescence combined with DAPI staining for nuclei (scale bar: 50 μm). **F** The mRNA levels of CD68 and Arg-1 were detected by qRT-PCR. **G**, **H** The protein expression of Iba-1, CD68, Arg-1 were detected by western blot and quantified by Image J software. The data are presented as the mean ± SD. *n* = 5. NS not significant, *P < 0.05, **P < 0.01.

Under pathophysiological conditions, activated microglia are defined as classic (proinflammatory; M1-like) or alternative (anti-inflammatory or protective; M2-like) [21]. M1-like microglia secrete proinflammatory cytokines and aggravate inflammation and tissue damage, while M2-like microglia have an anti-inflammatory effect [22, 23]. Since itaconate can reduce the inflammation of microglia induced by LPS, we further examined how the polarization of microglia changes under the influence of itaconate. After the stimulation with LPS, there was an increase in the expression of Iba-1, which is a classic M1 type microglia marker (Fig. 6A, B). After adding 4-OI, the high expression of Iba-1 was inhibited (Fig. 6G). The addition of itaconate suppressed the expression of iNOS, COX2, and IL-6. All these results indicated that itaconate can inhibit M1 polarization in microglia. Moreover, the microglia became amoebic in appearance shape under the stimulation of LPS, indicating M1 polarization, and 4-OI reduced the proportion of microglia that change morphologically (Fig. 6C) The results of immunofluorescence, qRT-PCR, and western blot analysis showed that 4-OI inhibits the increase in CD68 and promotes the expression of Arg-1 (Fig. 6D–H). CD68 is a panmacrophage marker and high expression of CD68 is associated with persistent inflammation, while Arg-1 is an M2 microglia/macrophage marker [24–26]. Collectively, these results suggested that itaconate can inhibit M1 polarization and promote M2 polarization in microglia.

### Itaconate suppresses M1 microglia-induced apoptosis of PC12 cells

**Fig. 7:**
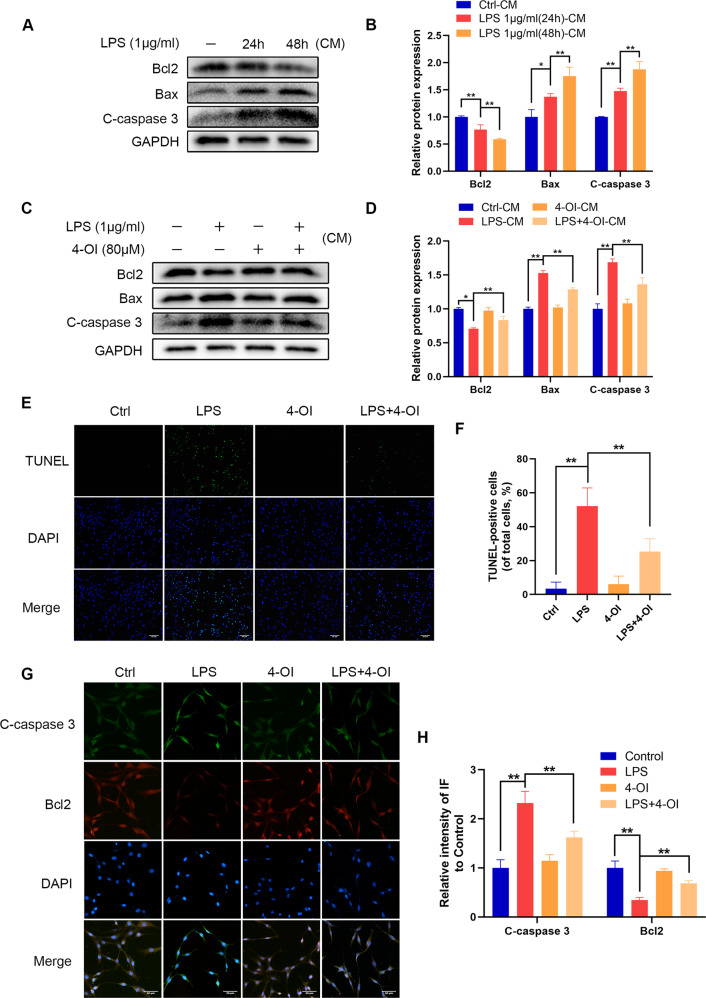
4-OI reduces PC12 cells damage caused by M1 microglia. **A**, **B** The protein levels of Bcl2, Bax, and Cleave-caspase3 in PC12 cells were detected by western blot and quantified by Image J software. **C**, **D** The protein levels of Bcl2, Bax, and Cleave-caspase3 in PC12 cells after treating with or without 4-OI. **E**, **F** The apoptosis level of PC12 cells was detected by TUNEL staining (scale bar: 50 μm). **G**, **H** The Cleave-caspase3/Bcl2 was detected by immunofluorescence combined with DAPI staining for nuclei (scale bar: 50 μm). The data are presented as the mean ± SD. *n* = 5. *P < 0.05, **P < 0.01.

During spinal cord injury, M1 microglia produce and release inflammatory mediators that damage neurons. In previous studies, we showed that itaconate can reduce the inflammatory mediators produced by microglia stimulated by LPS. To further investigate whether this effect of itaconate can protect neurons from damage, we used conditioned medium (CM) treatment and transwell co-culture system to study the effects of microglia on PC12 cells, which are widely used to study the neuroprotective effects of compounds [27, 28]. CM was collected from each set of differently treated microglia cultures. PC12 cells were stimulated by CM collected from LPS-treated microglia and showed increased levels of apoptosis at 24 and 48 h, according to the results of the western blot analysis (Fig. 7A, B). However, under the stimulation of the CM with 4-OI, the protein levels of Bax and Cleave-caspase3 were decreased, and the expression of Bcl2 was increased in PC12 cells (Fig. 7C, D). Bcl2 has the effect of resisting cell apoptosis, and the increase in Cleave-caspase3 and Bax expression indicated an increase in the level of apoptosis [29]. These results suggested that 4-OI protected the PC12 cells from apoptosis. Next, we performed TUNEL staining and immunofluorescence double staining of Cleave-caspase3 and Bcl2 on PC12 cells after co-culture of microglia. After 48 h of co-cultivation with 4-OI-treated microglia, the proportion of apoptotic PC12 cells decreased significantly, the expression of Cleave-caspase3 decreased, and the expression of Bcl2 increased (Fig. 7E–H). To sum up, these results demonstrated that the anti-inflammatory effect of itaconate can reduce neuronal apoptosis induced by M1 microglia.

### Itaconate suppresses inflammation through Nrf2

**Fig. 8:**
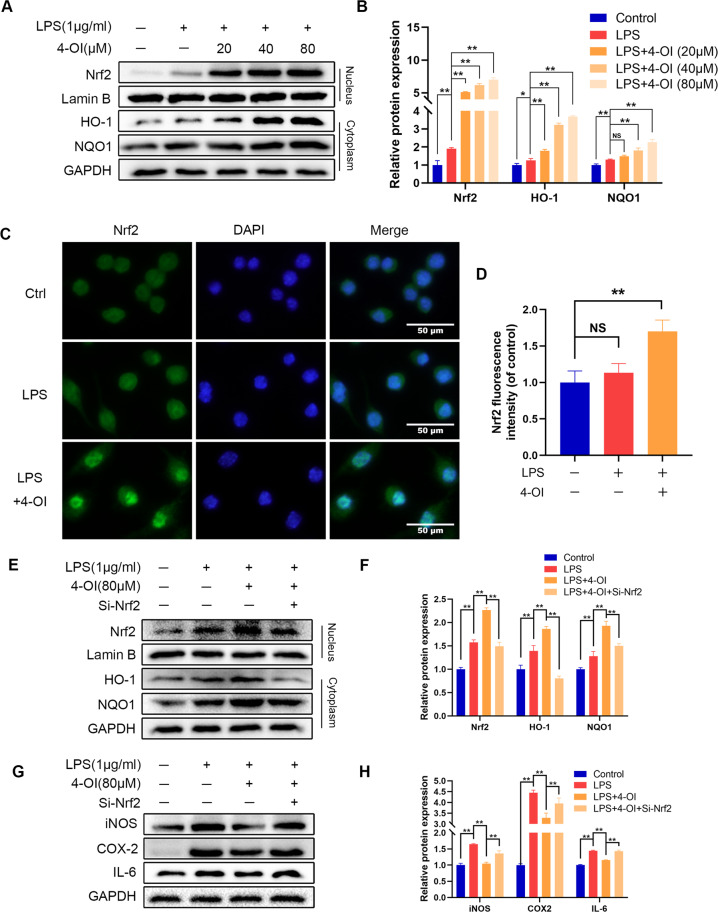
Effect of 4-OI on the Nrf2 pathway in LPS-treated microglia. **A**, **B** The translocation of Nrf2 into the nucleus and cytoplasm of HO-1 and NQO1 expression were detected by western blot and quantified by Image J software. **C**, **D** The Nrf2 was detected by immunofluorescence combined with DAPI staining for nuclei (scale bar: 50 μm). **E**, **F** After treatment with 4-OI, Nrf2 in the nucleus and HO-1 and NQO1 in the cytoplasm were detected with western blot. **G**, **H** The protein expression of iNOS, COX2, IL-6 were detected by western blot. The data are presented as the mean ± SD. *n* = 5. NS not significant, *P < 0.05, **P < 0.01.

Our study found that microglia overexpressing Irg-1 can activate the Nrf2 pathway. Next, we tested whether 4-OI as an exogenous itaconate can also activate the Nrf2 pathway. As shown in Fig. 8A, B and Supplementary Fig. S3E, F, the results of the western blot analysis showed that 4-OI could promote the nuclear import of Nrf2 and significantly increase the expression of HO-1 and NQO1, and this promotion effect increased with the increase in the concentration of 4-OI. In addition, the results of immunofluorescence staining also showed that the entry of Nrf2 into the nucleus of microglia increased after 4-OI treatment (Fig. 8C, D). Moreover, after treatment with Nrf2 siRNA, accompanied by the inhibition of the Nrf2 pathway, the level of inflammatory indicators was significantly higher than that in the 4-OI treatment group (Fig. 8E–H and Supplementary Fig. S3G, H). These results suggested that itaconate can also activate the Nrf2 pathway and relies on the Nrf2 pathway to exert its anti-inflammatory effects.

### Itaconate improves functional recovery after SCI in mice

**Fig. 9:**
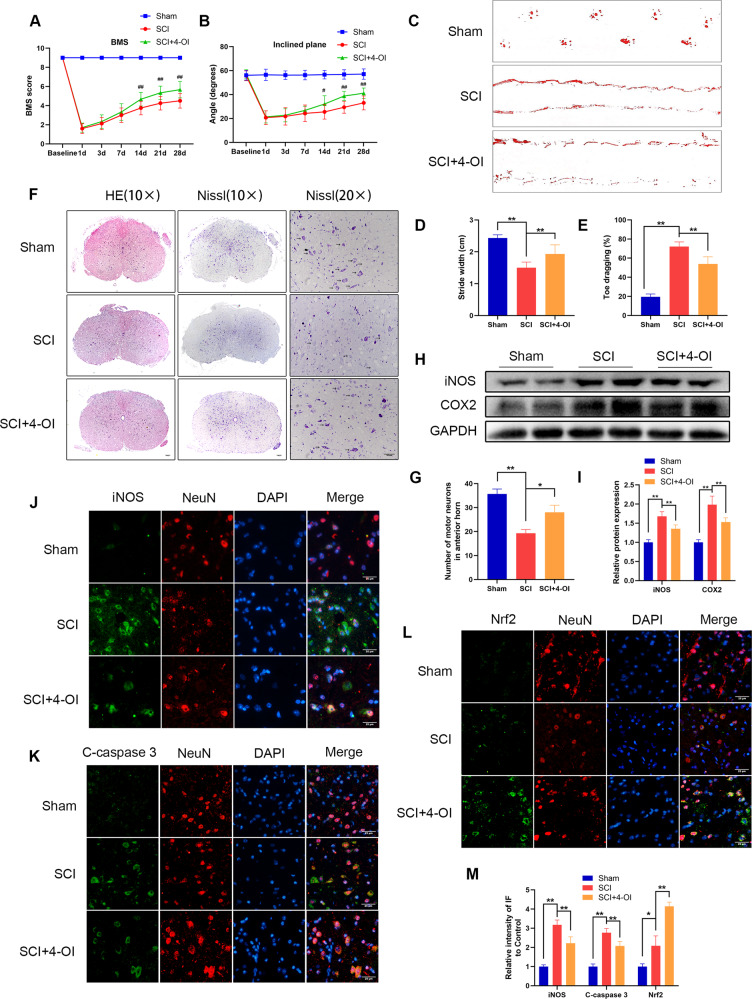
4-OI reduces inflammation after spinal cord injury in mice and accelerates functional recovery. **A**, **B** Basso mouse scale (BMS) and inclined plane test for the indicated groups at days 1, 3, 7, 14, 21, and 28 (*n* = 8–12). ^#^*P* < 0.05, ^##^*P* < 0.01 relative to the SCI group. **C** Photos of mice footprints at day 28 after SCI. **D**, **E** Quantification of stride width and toe dragging in the footprint analysis. **F** The transected spinal cord sections from each group were analyzed at day 28 by HE staining and Nissl staining. The black arrows indicate motor neurons (scale bar: 100 μm and 50 μm). **G** Quantitative analysis of Nissl positive motor neurons in the anterior horn of spinal cord in each group. **H**, **I** The expression of iNOS and COX2 in the spinal cord tissues of each group were detected at day 28 by western blot and quantified by Image J software. **J**–**M** The iNOS/NeuN, Cleave-caspase3/NeuN and Nrf2/NeuN in spinal cord injury were detected at day 28 by immunofluorescence combined with DAPI staining for nuclei (scale bar: 25 μm). The data are presented as the mean ± SD. *n* = 6 (except for BMS and inclined plane test). *P < 0.05, **P < 0.01.

In vitro experiments showed that itaconate can activate the Nrf2 pathway and protect the vitality of PC12 cells. In the in vivo experiment, SCI-modeled mice from the SCI+4-OI group were intraperitoneally injected with 4-OI (50 mg/kg) once every 2 days after surgery, while the other mice were intraperitoneally injected with the same volume of DMSO solution. According to BMS scores and the results of the inclined plane test and footprint analysis, the motor function of mice treated with 4-OI recovered better than that of the SCI mice (Fig. 9A–E). In addition, H&E staining and Nissl staining also showed that the spinal cord recovered and the number of neurons increased after 4-OI treatment (Fig. 9F, G). At the molecular level, after protein extraction from the spinal cord of the mice, the results of western blot analysis results indicated that the expression levels of iNOS and COX2 in SCI mice decreased after intraperitoneal injection of 4-OI (Fig. 9H, I). Moreover, immunofluorescence staining of spinal cord tissue showed that 4-OI decreased the level of inflammation and apoptosis of neurons and increased the expression of Nrf2 (Fig. 9J–M). Thus, these findings suggested that itaconate can not only improve functional recovery but also reduce tissue disorder and neuron loss after traumatic SCI.

## Discussion

Neuroinflammation, especially the activation of microglia and macrophages, is a sign of spinal cord injury [30]. However, microglia are a double-edged sword in the process of nerve recovery from spinal cord injury [9, 30]. Activated microglia can swallow and remove cell debris and release neurotrophic factors [31, 32]. On the other hand, over-activated microglia often release a large number of proinflammatory mediators, further aggravating the damage to nerve cells [33, 34]. Several studies have shown that regulating the metabolism and inflammation of microglia is beneficial to the survival of nerve cells [35–37]. In our study, we found that Irg-1 is associated with the progression of spinal cord injury. Overexpression of Irg-1 and exogenous supplementation of its regulated metabolite itaconate can inhibit inflammation of microglia, thereby protecting nerve cells. In vivo, Irg-1/itaconate reduces inflammation levels and promotes functional recovery function in mice after spinal cord injury.

Irg-1 is a mitochondria-related enzyme-coding gene that has various functions such as regulation of infection, inflammation, metabolism, etc. [14, 20, 38]. Irg-1 is one of the genes whose expression levels are significantly increased in mouse microglia under proinflammatory conditions such as bacterial infections [11–13, 39]. In vivo and in vitro, we found that the expression of Irg-1 was related to the progression of spinal cord injury. After knocking out or overexpressing Irg-1 with a lentivirus, our results showed that Irg-1 can inhibit microglia inflammation caused by LPS. In the in vivo experiments, after overexpression of Irg-1, the inflammation level decreased in mice after spinal cord injury and the motor function recovered faster. However, although Irg-1 can protect the spinal cord from injury in mice, its protective effect is insufficient without intervention since the expression peak time of Irg-1 is relatively short.

Itaconate, a metabolite synthesized by the enzyme encoded by Irg-1, is increased in LPS-activated macrophages and has been shown to regulate metabolism, resist inflammation, and play an immunomodulatory role in mammals [12, 16, 40, 41]. Since Irg-1 can protect the spinal cord from injury, we investigated whether itaconate has the same protective effect and can compensate for the lack of Irg-1 in the pathological process. Therefore, we used 4-OI, a cell-permeable itaconate derivative, which can mimic the effect of endogenous itaconate better than other derivatives [20]. From our experiments, we found that like Irg-1, itaconate can inhibit microglia inflammation and promote the functional recovery of mice after spinal cord injury. Itaconate exerts its anti-inflammatory effects by regulating the polarization level of microglia and activating the Nrf2 pathway.

In the pathological process of spinal cord injury, the polarization level of microglia is closely related to its function [9, 42]. Classically activated (M1) microglia induced by LPS release destructive proinflammatory mediators [37, 43]. In contrast, alternatively activated (M2) microglia and macrophages remove cell debris through phagocytosis and release many protective and nutritional factors, which might have neuroprotective functions [44]. Generally, promoting the M2 phenotype and inhibiting the M1 phenotype is beneficial to the recovery and function of nerves after spinal cord injury [45]. After using itaconate, the marker Iba-1 of the M1 phenotype decreased, while the marker Arg-1 of the M2 phenotype increased, which indicated that itaconate can promote M2 polarization of microglia and inhibit M1 polarization. In addition, through the co-culture system, we evaluated the effect of itaconate-treated microglia on nerve cells. The results showed that itaconate reduces neuronal apoptosis caused by M1 microglia.

Nrf2 is a transcription factor that regulates the cellular defense against inflammation, oxidative stress and apoptosis [46–48]. At rest, Nrf2 binds to seaweed-like ECH-related protein 1 (Keap1)-Cullin3-Rbx1 to maintain its stability [49]. After activation, the Nrf2 protein separates from Keap1 and translocates to the nucleus. Subsequently, Nrf2 integrates with ARE and then promotes transcription of antioxidant and detoxifying genes, including HO-1 and NQO1 [50, 51]. In our study, after overexpression of Irg-1 and the use of itaconate, the expression of Nrf2 in the nucleus and HO-1 and NQO1 in the cytoplasm increased, indicating the activation of the Nrf2 pathway. Furthermore, the application of siRNA to knockdown Nrf2 showed that the protective effect of Irg-1 and itaconate was significantly reduced. These results provide evidence that Irg-1 and itaconate can activate the Nrf2 pathway and have a protective role accordingly.

Although we did not evaluate the sensory function in in vivo study, it does not mean that the effects of Irg-1/itaconate are motor neuron-specific. The results of Nissl staining in either Irg-1 overexpression group (Fig. 4H) or itaconate treatment group (Fig. 9F) showed an increased number of neurons. As the Nissl staining is not specific to sensory neurons or motor neurons, the increased number of neurons may due to both sensory neurons and motor neurons. More neurons may lead to improved functions, thus the increased number of sensory neurons and motor neurons may lead to both improved motor function and sensory function.

Itaconate was systemically administrated in our study, which implies that itaconate may exert its effects not only on neurons, but also on other cells. According to the reported literature, itaconate can reduce the accumulation of neutrophils and the secretion of inflammatory factors in acute lung injury induced by LPS [52]. In addition, Irg-1/itaconate can also inhibit the activation of NLRP3 inflammasomes, thereby combating macrophage inflammation and bacterial infections [15, 53, 54]. Based on these results, we speculate that itaconate can also promote the recovery of spinal cord injury by regulating the function of macrophages, and neutrophils, which of cause needs further validation.

Collectively, we revealed that the expression of Irg-1 is related to the progression of spinal cord injury. Under inflammatory conditions, overexpression of Irg-1 or supplementation of itaconate can activate the Nrf2 pathway to reduce inflammation of microglia, thereby alleviating neuronal damage caused by M1 microglia. Moreover, in vivo experiments demonstrated that overexpression of Irg-1 and application of itaconate can reduce the inflammation level of spinal cord injury in mice and promote functional recovery after injury. Therefore, we proposed that the activation of Irg-1 and the application of itaconate might be a promising treatment strategy for SCI.

This study had several limitations and open questions. In the experiments, we used LPS-induced polarized microglia and neural cell line PC12 cells to simulate the effects of microglia on neurons in spinal cord injury [55, 56]. However, the changes in the microenvironment after spinal cord injury are extremely complex, and the use of these two types of cells cannot completely simulate the changes in the microenvironment after spinal cord injury. In addition, the peak of Irg-1 expression after spinal cord injury in mice was shorter than the peak of Irg-1 in the in vitro experiments. This could be due to the negative feedback inhibition of Irg-1 after itaconate expression increases, and this negative feedback effect may affect Irg-1/itaconate to better exert its effect. Furthermore, the method to apply Irg-1/itaconate in clinical treatment also requires further investigation.

## Materials and methods

### Reagents and antibodies

LPS (#L3129) were purchased from Sigma-Aldrich (WI, USA), and 4-OI (#HY-112675) was purchased from MCE (NJ, USA) and dissolved in DMSO (Solarbio, #D8371, Beijing, China). The primary antibody against iNOS (18985-1-AP), Bax (50599-2-Ig), Nrf2 (16396-1-AP), Lamin B (66095-1-Ig), GAPDH (60004-1-Ig), IL-6 (66146-1-Ig), NQO1 (67240-1-Ig), Arg-1 (66129-1-Ig) and CD68 (28058-1-AP) were obtained from ProteinTech (Wuhan, China). The primary antibodies against HO-1 (#43966), COX2 (#12282), Irg-1 (#17805), Iba-1 (#17198), and Cleaved Caspase3 (#9661) were purchased from CST (MA, USA), and the primary antibodies against NeuN (ab104224), Bcl2 (ab196495) and Alexa Fluor 488-labeled and Alexa Fluor 594-labeled goat anti-rabbit/mouse secondary antibodies were purchased from Abcam (Cambridge, UK). Finally, 4′,6-diamidino-2-phenylindole (DAPI) was purchased from Yeasen Biochemical (Shanghai, China).

### Mouse spinal cord injury model

Briefly, after anesthesia with 2% (w/v) pentobarbital (40 mg/kg) intraperitoneally, C57BL/6 female mice (6–8 weeks old) were placed on a constant temperature electric blanket that was maintained at 37 °C during the entire SCI modeling process. Laminectomy was performed at the T9–T10 level to expose the dorsal surface of the spinal cord without damaging the dura mater. Then, the mice with the exposed spinal cord were fixed to the spinal cord weight drop, and a 5 g weight was released from a height of 30 mm to hit the exposed spinal cord to induce moderate spinal cord injury according to the manufacturer’s instructions (W.M. KECK+, Model+III, USA) [57]. Then, the muscle and skin were sutured in layers with 4-0 silk and needle. After SCI, the mice received manual urination three times a day until the bladder reflex was established. Sham-operated mice received anesthesia and laminectomy but did not experience spinal cord injury.

### Lentiviral intrathecal injection

The LV-shIrg-1 and LV-Irg-1 were purchased from GeneChem (Shanghai, China, Supplementary Table 1). Fifteen minutes after causing moderate spinal cord injury, mice were injected intrathecally with lentivirus. Inject 0.5 μl at each of the two points on the upper and lower planes of the striking position. The muscles and skin were sutured layer by layer, and the mice were placed on an electric blanket at 37 °C until they were awakened to carry out follow-up experiments (Supplementary Fig. S1A).

### Drug administration

From the first day after surgery, 4-OI was injected intraperitoneally at a concentration of 50 mg/kg until the mice were sacrificed, and the frequency was once every 2 days (Supplementary Fig. S1B). The sham group and SCI group were injected with the same dose of normal saline.

### Behavior assessment

#### The BMS score

After 1, 3, 7, 14, 21, and 28 days of the spinal cord injury, the mice were placed in an open area, allowed to move freely for 5 min, and observed for 4 min. According to the degree of the posterior ankle joint mobility, coordination, paw posture, trunk stability, and tail posture, the BMS scoring system was used to score the activity of mice from 0 to 9 points [58].

#### Inclined plane test

The oblique board test was used to evaluate the strength of the hind limbs of mice. The mouse to be tested was placed on an inclined plane with a rubber surface. After the angle of inclination was increased to a certain degree, the mouse fell off the plane. The maximum angle at which the mouse could stay on the inclined plane for 5 s without falling was recorded. Each mouse was evaluated three times, with an interval of 1 min between each evaluation [59].

#### Footprint analysis

The hind limbs of the mice in different groups of mice were immersed in red ink, and the mice were allowed to walk on a white track to obtain their movement trajectory. Footprints are quantitatively analyzed by stride width and toe dragging. Stride width is the distance between the footprint of the hind limbs and toe dragging is the ratio of total hindlimb dragging length and walking length [60, 61]. All behavioral evaluations are were performed by individuals who were unaware of the experimental groupings.

### Tissue preparation

After euthanizing the mice at a specific time point, transcardial perfusion with phosphate-buffered saline (PBS) was performed. The spinal cord was cut (1 cm) with the site of injury as the center, and immediately stored in a refrigerator at –80 °C for subsequent western blot analysis and qRT-PCR. For staining, the mice were perfused with PBS and 4% paraformaldehyde. After the spinal cord was cut, it was fixed with 4% paraformaldehyde overnight, rinsed with water, and immersed in 75%, 85%, 95%, and 100% ethanol solution for 90 min according to the concentration gradient. After soaking in xylene twice for 20 min each time, the sample was soaked in paraffin wax (melting point: 54 °C) for 1 h three times. Finally, the sample was soaked in paraffin wax (melting point: 60 °C) to completely dehydrate it. For embedding, the spinal cord tissue was first cut into two sections with the point of injury as the center, with the injured side facing down, and then, it was embedded in a specific mold with paraffin wax (melting point: 60 °C). The wax blocks were cut into ten tissue sections with a thickness of 5 μm for H&E staining, Nissl staining, and immunofluorescence staining.

### Cell culture and drug administration

Mouse microglial cell line (BV2 cell) and PC12 neuronal cell line were purchased from Procell Life Science & Technology (Wuhan, China). The cells were maintained in high glucose Dulbecco’s modified Eagle’s medium (DMEM) supplemented with 10% fetal bovine serum, 100 units/ml penicillin, and 100 mg/ml streptomycin. They were maintained in a humidified air environment with 5% CO_2_ at 37 °C. For injury stimulation in vitro, microglia were treated with LPS. And 4-OI was added to the cells based on the results of CCK-8 and the experimental design.

To investigate the relationship between microglia and PC12 cells, we used two methods: transwell co-culture system (Corning, 0.4-μm well, USA) and CM treatment (Supplementary Fig. S2). In the transwell co-culture system, microglia were cultured in the upper chamber of the transwell six-well plate, and PC12 are cultured in the six-well plate; the two were cultured separately. After the microglia were treated with LPS or 4-OI, the upper chamber containing the microglia was transferred to a six-well plate and co-cultured with PC12 cells for 48 h, and then, the PC12 cells were detected by immunofluorescence and TUNEL staining. Regarding the CM treatment, the treated microglia culture medium was collected, centrifuge at 3000 rpm for 5 min to remove the cell debris, and used for ELISA. The remaining part of the medium was used as the CM, diluted with an equal volume of serum-free medium and added to the culture dish of PC12 cells for western blot analysis.

### Cell Counting Kit-8

Microglia were seeded in 96-well plates at a density of 8000 per well. When the cell density reached 50%, they were washed with PBS three times, and 100 µl of medium containing different concentrations of drugs were added and incubated for 24 h. After washing three times with PBS, 100 μl of DMEM containing 10 μl of CCK-8 solution was added to each well, and the plate was incubated for another 2–4 h. The absorbance of each well was measured at 450 nm using a microplate reader.

### Separation of cytoplasmic and nuclear proteins

Nuclear and Cytoplasmic Protein Extraction Kit (P0027, Beyotime Biotechnology, Shanghai, China) was used to separate nuclear protein and cytoplasmic protein. The experimental operation process is carried out according to the instructions. Briefly, first wash the cells in the petri dish with PBS, then scrape the cells with a spatula and collect them in a centrifuge tube. After aspirating the supernatant, add 200 μl PMSF-added cytoplasmic protein extract A. Vortex at the highest speed for 5 s, after 15 min in an ice bath, add 10 μl cytoplasmic extract B. Vortex again at the highest speed for 5 s, centrifuge at 12,000–16,000 g at 4 °C for 5 min, and draw the supernatant to obtain the cytoplasmic protein. For precipitation, add 50 μl of nucleoprotein extractant with PMSF, vortex at the highest speed for 30 s, and vortex intermittently for half an hour after an ice bath. After centrifugation at 12,000–16,000 g for 10 min at 4 °C, the supernatant is the nuclear protein.

After detecting its concentration by BCA kit and adding reagents to balance, use western blot to detect.

### Western blot analysis

The cells and spinal cord tissue (1 cm long) were lysed with RIPA containing 1% PMSF, and the protein was collected after ultrasound treatment and ultracentrifugation at 12,000 rpm for 30 min. After detecting the protein concentration with BCA analysis tool kit (Beyotime, Shanghai, China), the protein concentration was fixed to a specific level (80 μg in vivo and 40 μg in vitro). The proteins were separated on 10–12.5% sodium dodecyl sulfate–polyacrylamide gel electrophoresis and transferred to polyvinylidene fluoride membranes. After blocking with 5% non-fat milk in TBST (Tris-buffered saline with 0.1% Tween-20), the membranes were incubated overnight at 4 °C with primary antibodies. The membranes were washed thrice with TBST for 3 min each time and incubated with HRP-conjugated IgG secondary antibody at room temperature for 1 h. Finally, after washing three times with TBST, the membrane blot was detected using the Chemi DocXRS + imaging system (Bio-Rad, USA) with a super-sensitive ECL chemiluminescence kit (Epizyme Biomedical Technology, Shanghai, China), and quantitative analysis was performed using the Image J software [62]. The concentration of antibody used in western blot is shown in Supplementary Table 2.

### TUNEL staining

The TUNEL staining is used for measuring apoptotic DNA fragmentation. PC12 cells were seeded on slides in a six-well plate and allowed to attach for 24 h. After co-culture of microglia and PC12 cells for 48 h, the PC12 cells were washed gently with PBS three times and fixed with freshly prepared 4% paraformaldehyde for 30 min before being incubated with 0.1% Triton X-100 for 5 min. Then, the cells were stained with the TUNEL Apoptosis Detection Kit (Yeasen Biochemical, Shanghai, China) for 30 min at 37 °C according to the manufacturer’s instructions, and the nuclei were stained with DAPI. The images were captured by a Nikon ECLIPSE Ti microscope (Nikon, Tokyo, Japan).

### Lentivirus and siRNA transfection

For transfection, microglia were inoculated at a density of 40–60% and infected with lentivirus at a multiplicity of infection (MOI) of 20. After 12 h of transfection, the medium was changed. Following cell confluence, the transfected microglia were passaged for further experiments. To suppress the expression of Nrf2, microglia (50–60% confluence) were transfected with Nrf2 small interfering RNAs (Si-Nrf2; RiboBio, China, Supplementary Table 3) according to the manufacturer’s protocol. The transfection efficiency was evaluated by western blot analysis.

### Quantitative real-time polymerase chain reaction

Cells were seeded in six-well plates in advance, and the cells were processed according to the experimental design. The cells and spinal cord tissue with Trizol to extract RNA according to the manufacturer’s instructions. Then, reverse transcriptase was used to synthesize cDNA. qRT-PCR were performed using a reaction volume of 10 μl, which included 5 μl of 2*SYBR Master Mix, 0.25 μl of each primer and 4.5 μl of diluted cDNA. The primer sequences are listed in Table 1. The qRT-PCR parameters included 5 s at 95 °C, 15 s at 95 °C, and 30 s at 60 °C; a total of 40 cycles were performed. The completion of the reaction depended on the CFX96Real-Time PCR system (Bio-Rad Laboratories, California, USA). Finally, these targeted mRNAs were normalized to GAPDH mRNA.

**Table 1:** Sequences of primers in qRT-PCR.

Primer name	Primer sequences
iNOS	5’-GGAGTGACGGCAAACATGACT-3’ (forward) 5’-TCGATGCACAACTGGGTGAAC-3’ (reverse)
COX2	5’-TGTGACTGTACCCGGACTGG-3’ (forward) 5’-TGCACATTGTAAGTAGGTGGAC-3’ (reverse)
GAPDH	5’-AAGAAGGTGGTGAAGCAGG-3’ (forward) 5’-GAAGGTGGAAGAGTGGGAGT-3’ (reverse)
Irg-1	5’-TGCTGCTGCGTCCAAGTTT-3’ (forward) 5’-GGGGCTTAGTCTGAGTGGC-3’ (reverse)
IL-6	5’-AGCCCACCAAGAACGATAG-3’ (forward) 5’-GGTTGTCACCAGCATVAGT-3’ (reverse)
Arg-1	5’-TTGGGTGGATGCTCACACTG-3’ (forward) 5’-GTACACGATGTCTTTGGCAGA-3’ (reverse)
CD68	5’-CCTCGCCTAGTCCAAGGTC-3’ (forward) 5’-GGATTCGGATTTGAATTTGGGCT-3’ (reverse)

### Immunofluorescence staining

Spinal cord slices (5 μm thick), after deparaffinization, were hydrated and washed three times with PBS. The cells were planted on a cover glass of a 12-well plate, fixed with 4% paraformaldehyde for 15 min, and washed three times with PBS for 3 min each time. Next, the sections and cells were incubated with 10% goat serum at 37 °C for 1 h, followed by incubation with the primary antibodies overnight at 4 °C in the same buffer.

After incubation with the primary antibodies, the sections were washed and incubated in secondary antibodies for 60 min at 37 °C. Finally, after washing three times with PBS, the slides were mounted with DAPI, the images were observed with a Nikon ECLIPSE Ti microscope (Nikon, Tokyo, Japan) and analyzed using Image J software [63]. The concentration of antibody used in immunofluorescence staining is shown in Supplementary Table 4.

### Hematoxylin and eosin (H&E) staining

The H&E staining kit was purchased from Solarbio (G1120, Beijing, China). The staining was performed according to the manufacturer’s instructions. First, the prepared tissue sections (5 µm thick) were placed in an oven at 62 °C for 30 min and soaked in xylene for 15 min twice. After dewaxing, the tissue sections were soaked in 100%, 95%, 85%, and 75% ethanol solution for 3 min, respectively, for hydration. The tissue sections were washed with distilled water for 5 min and dyed with the hematoxylin staining solution for 5 min. The tissues were washed again with distilled water for 5 min and differentiated with the differentiation solution for 2 min. After washing again for another 5 min, the tissue sections were dyed with eosin staining solution for 2 min. Finally, the tissue sections were successively soaked in 75–100% gradient ethanol solution for 3 min to complete the dehydration process. The slides were rinsed with xylene for 1 min, and were sealed with neutral resin. The images were captured with a Nikon ECLIPSE Ti microscope (Nikon, Tokyo, Japan).

### Nissl staining

The Nissl staining kit was purchased from Solarbio (G1430, Beijing, China). After the tissue sections were deparaffinized and hydrated, they were immersed in tar violet staining solution and stained at 56 °C for 1 h. They were then washed with deionized water for 5 min and differentiated with Nissl differentiation solution for 2 min until the background turned colorless. Finally, the tissue sections were dehydrated and sealed with neutral resin for preservation. The images were captured with a Nikon ECLIPSE Ti microscope (Nikon, Tokyo, Japan). Nissl positive cells were visualized and counted to define the motor neurons in the area of anterior horns, based on previous criteria [64].

### Statistical analysis

The results are presented as the mean ± standard deviation (SD) of at least five independent experiments. Data were analyzed by performing a one-way analysis of variance (ANOVA) followed by Dunnett’s post hoc test for comparison between control and treatment groups. Comparisons at multiple time points were performed by a two-way repeated-measures ANOVA with Bonferroni post hoc test. In case of cell line experiments, *n* = xxx represents repeats. In vivo experiments, *n* = xxx represents the number of mice or the number of spinal cord tissue. Differences between the groups were considered to be statistically significant at *P* < 0.05.

## Supplementary information

Checklist

Supplementary materials and methods

Supplementary material for the original image of WB

## Data Availability

The authors confirm that the data supporting the findings of this study are available within the article and its Supplementary Material. Raw data that support the findings of this study are available from the corresponding author, upon reasonable request.
