# Supplementary material for: Immune-responsive gene 1/itaconate activates nuclear factor erythroid 2-related factor 2 in microglia to protect against spinal cord injury in mice
Source: Cell Death Dis. 2022 Feb 10;13(2):140. doi: 10.1038/s41419-022-04592-4 (PMC8831631; doi:10.1038/s41419-022-04592-4)
Supplement: Supplementary file 2 — Supplementary materials and methods [file 41419_2022_4592_MOESM2_ESM.docx]

**Supplementary Information**

**for**

**Immune-responsive gene 1/itaconate activates nuclear factor erythroid 2-related factor 2 in microglia to protect against spinal cord injury in mice**

Libin Ni et al.

**Supplementary materials**


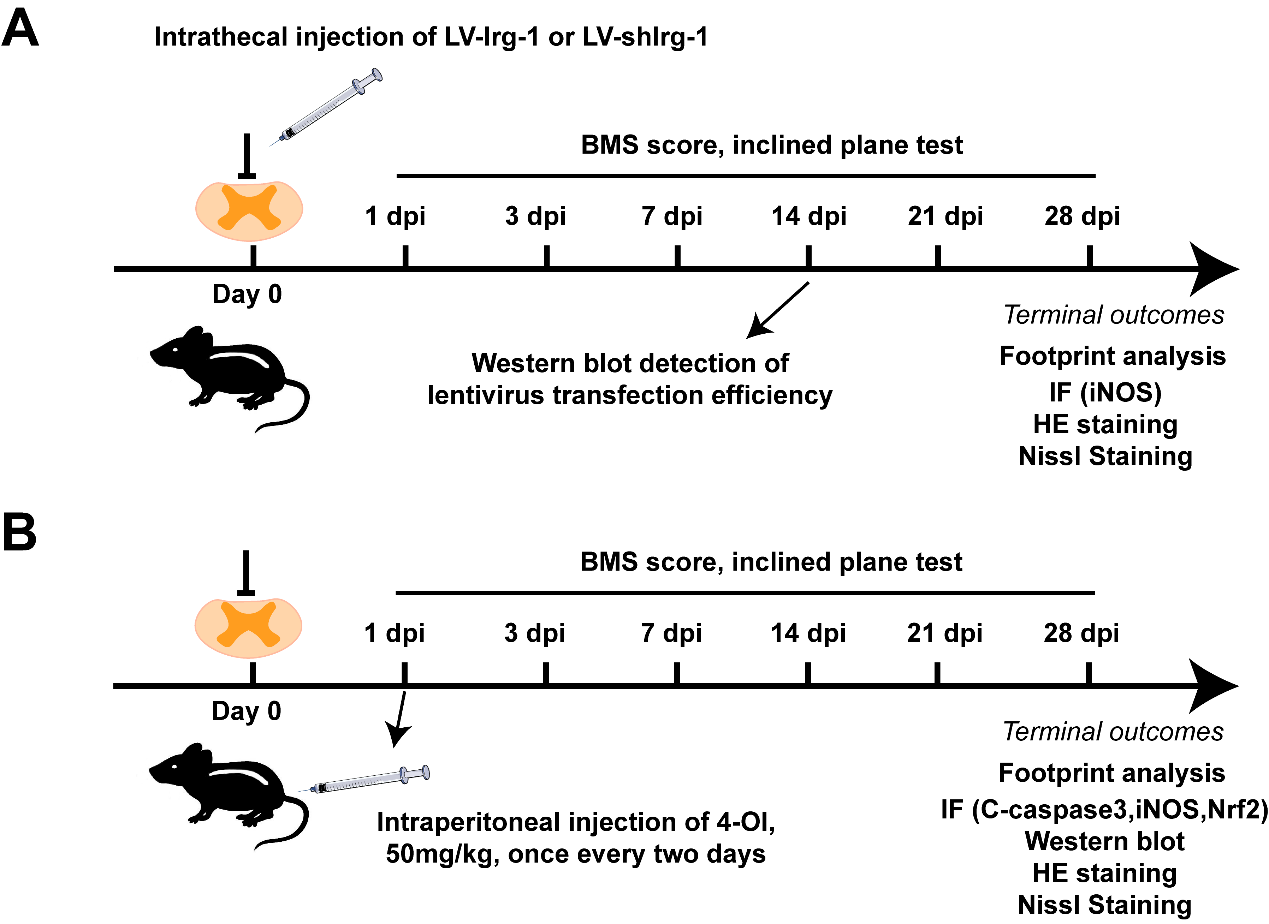


**Figure S1. Schematic diagram of the research process of *in vivo* experiments in mice. (A)** Experimental procedures related to lentivirus up-regulation or knock-out of Irg-1. **(B)** Experimental procedures related to exogenous supplementation of itaconate.


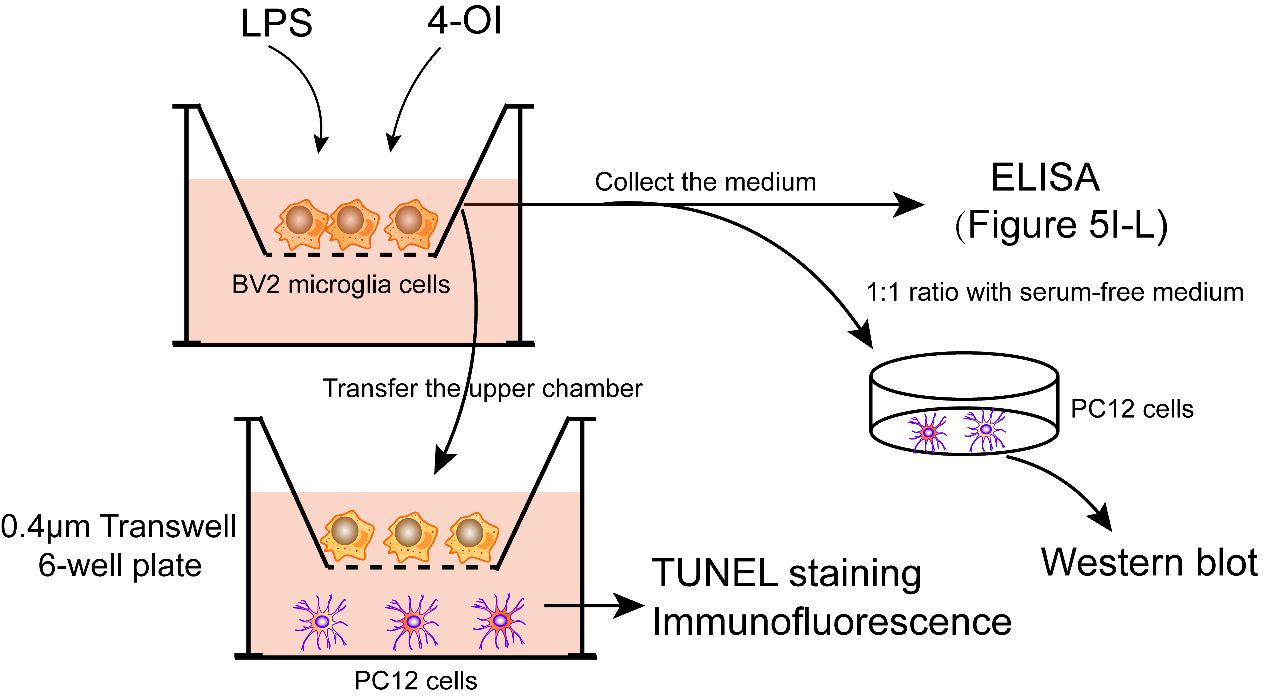


**Figure S2. Schematic diagram of the research process of the effect of microglia on PC12 cells.** We used two methods to carry out related experiments. PC12 cells co-cultured with microglia through a 0.4μm transwell 6-well plate are used for TUNEL staining and immunofluorescence staining, while PC12 cells treated with conditioned medium are used for western blot detection.


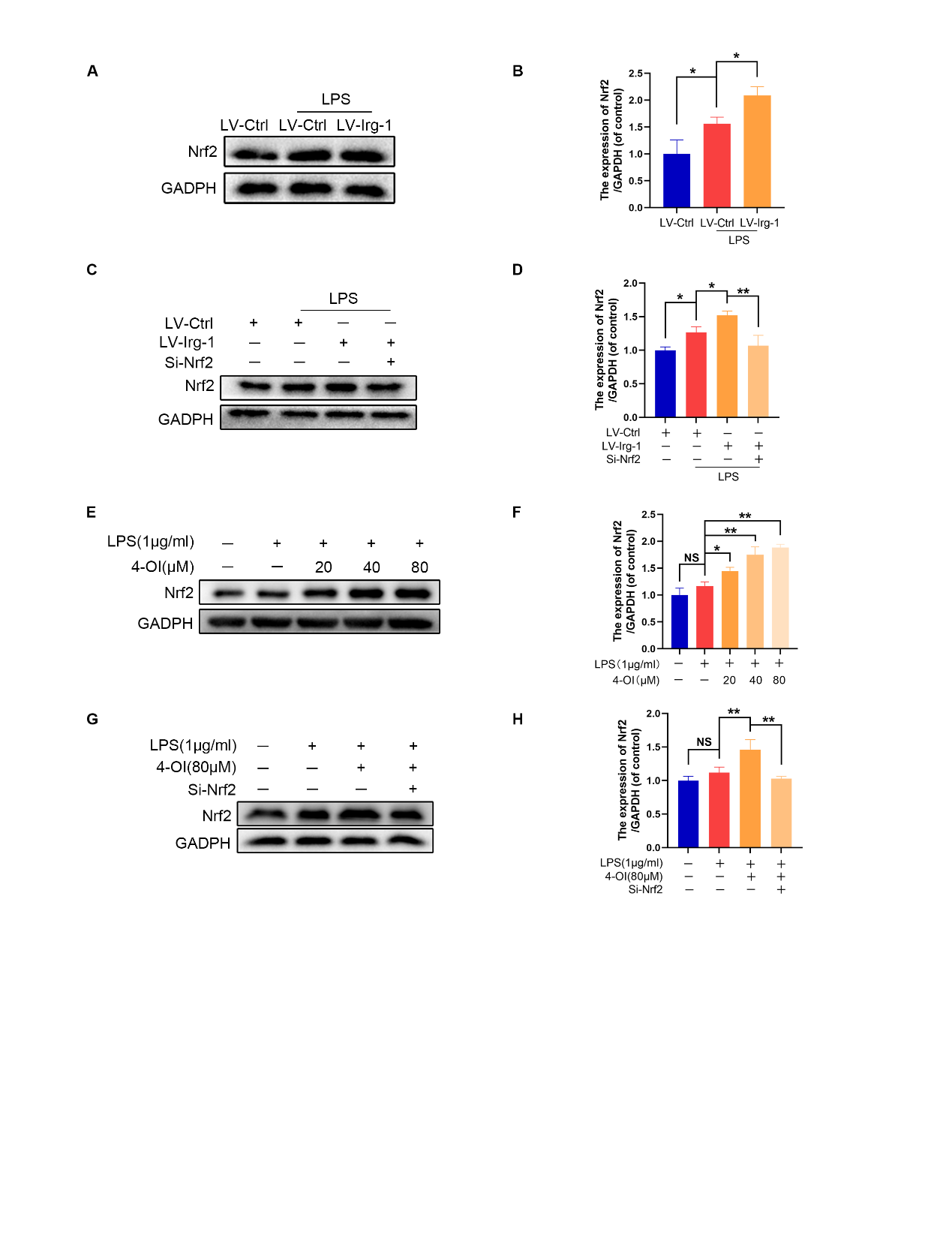


**Figure S3. Nrf2 expression in whole cells. (A-H)** The protein expression of Nrf2 was detected by western blot and quantified by Image J software. The data are presented as the mean ± S.D. n=5. NS stands for not significant, *P < 0.05, **P< 0.01.

**Supplementary** **Table 1 for the information of lentivirus**

|  | Component order | Titer (TU/mL) |
| --- | --- | --- |
| LV-Irg-1  LV-shIrg-1 | Ubi-MCS-3FLAG-CBh-gcGFP-IRES-puromycin hU6-MCS-CBh-gcGFP-IRES-puromycin | 3.5E+9  5E+8 |

**Supplementary Table 2 for the concentration of antibody used in western blot**

| Antibody | Concentration |
| --- | --- |
| Irg-1  iNOS  COX2  IL-6  Nrf2  HO-1  NQO1  Iba-1  CD68  Arg-1  Bcl2  Bax  Cleave-caspase3  Lamin B  GAPDH | 1:1000  1:1000  1:1000  1:800  1:800  1:1000  1:800  1:800  1:1000  1:1000  1:1000  1:1000  1:500  1:2500  1:2500 |

**Supplementary Table 3 for the information of Si-Nrf2**

|  | Target sequence |
| --- | --- |
| si-r-Nrf2_001  si-r-Nrf2_002  si-r-Nrf2_003 | CAAACAGAATGGACCTAAA  GCAAGAAGCCAGATACAAA  GGATGAAGAGACCGGAGAA |

**Supplementary Table 4 for the concentration of antibody used in immunofluorescence**

| Antibody | Concentration |
| --- | --- |
| Nrf2  iNOS  Iba-1  CD68  Arg-1  Cleave-caspase3  Bcl2 | 1:400  1:250  1:400  1:400  1:400  1:200  1:400 |
