# Supplementary material for: Immune-responsive gene 1/itaconate activates nuclear factor erythroid 2-related factor 2 in microglia to protect against spinal cord injury in mice
Source: Cell Death Dis. 2022 Feb 10;13(2):140. doi: 10.1038/s41419-022-04592-4 (PMC8831631; doi:10.1038/s41419-022-04592-4)

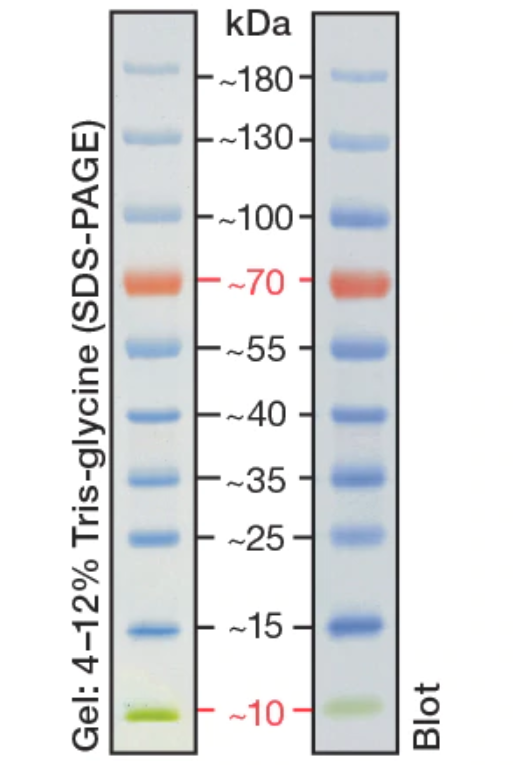

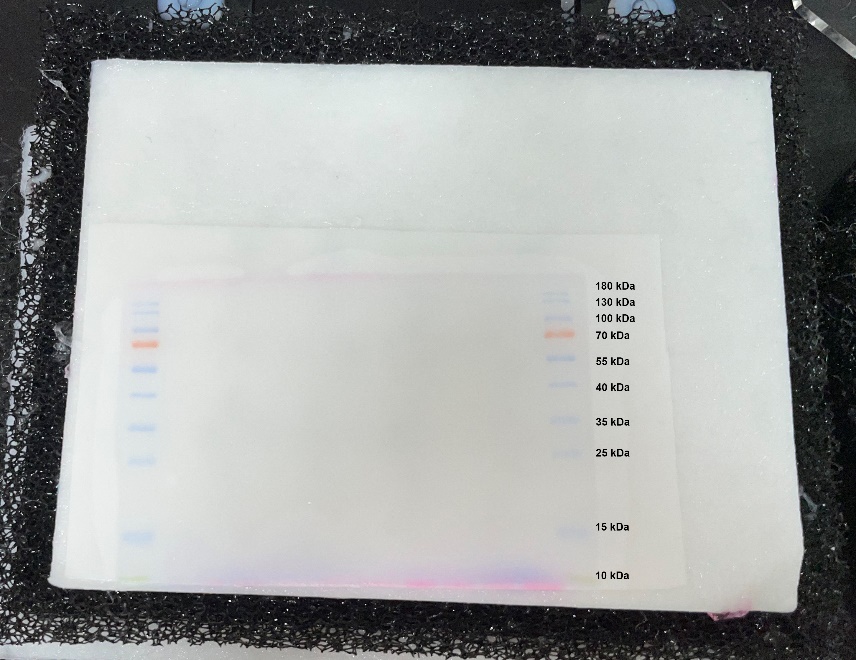


Marker was purchased from Thermo Fisher Scientific (26616, Shanghai, China). Its displayed molecular weight is as shown above. In our experiment, we used 10% or 12.5% ​​SDS-PAGE gel. After transferring to PVDF membrane (ISEQ00010, Merck KGaA, Darmstadt, Germany), the distribution of Marker under 12.5% SDS-PAGE gel is shown in the figure. Then, we will cut out the corresponding membrane according to the molecular weight of the target protein and the interval indicated by the Marker, and incubate with the corresponding target protein primary antibody. However, during the exposure process, since the Marker cannot be combined with the antibody and ECL exposure solution, it cannot be displayed in the image acquired by the Chemi DocXRS+ imaging system (Bio-Rad, USA).

The following is the full images of uncropped blots, and we have marked the molecular weight of the upper and lower edges of the membrane and the molecular weight of the target protein on the original image.

Figure 1D Irg-1 50kDa


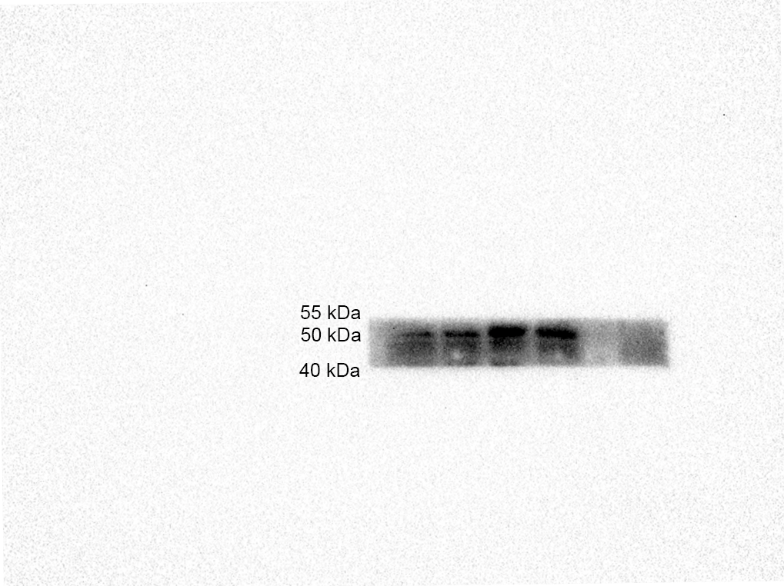


Figure 1D GAPDH 37kDa


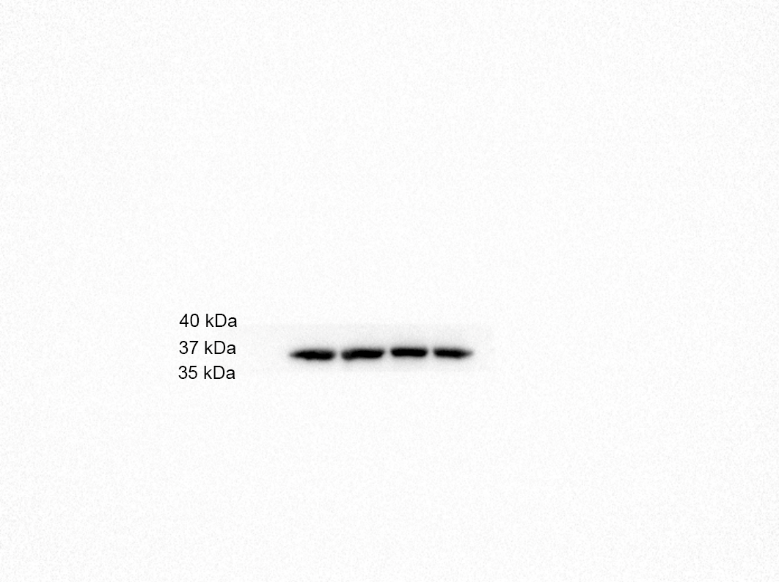


Figure 1F Irg-1 50kDa


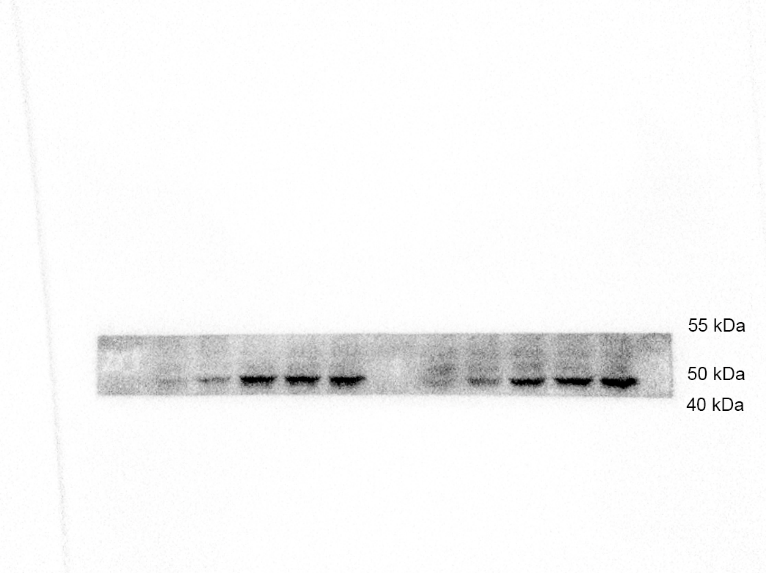


Figure 1F iNOS 131kDa


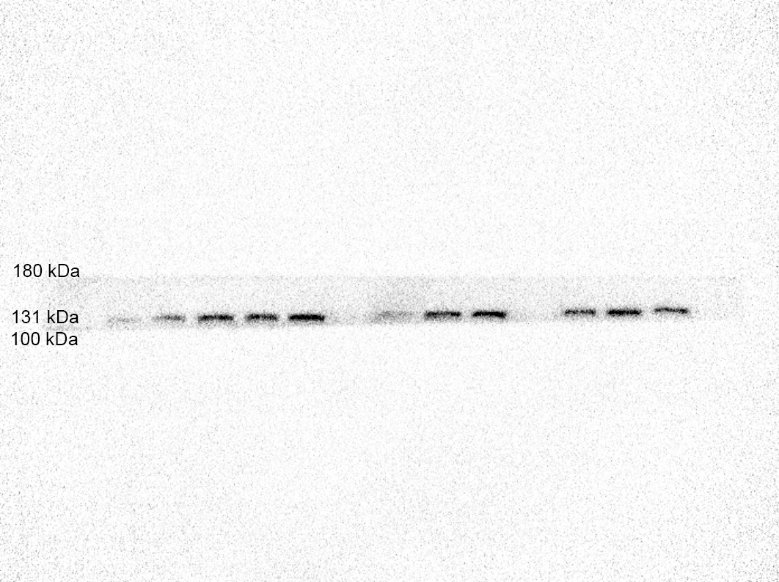


Figure 1F COX2 74kDa


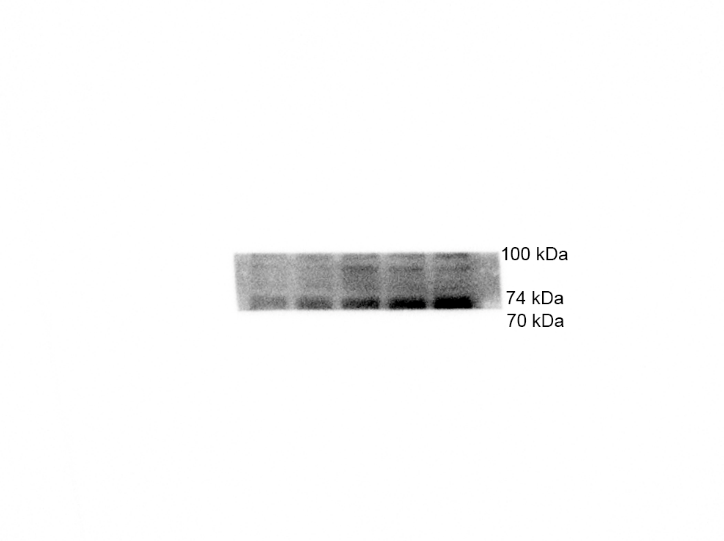


Figure 1F IL-6 24kDa


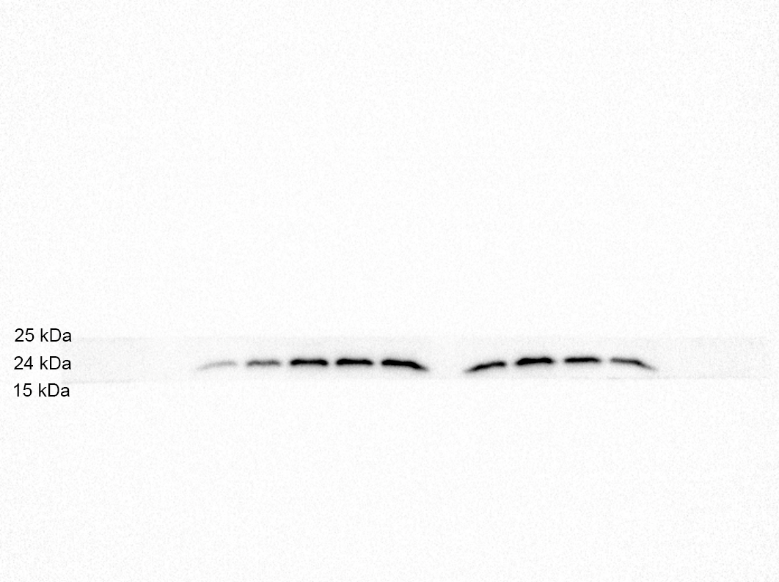


Figure 1F GAPDH 37kDa


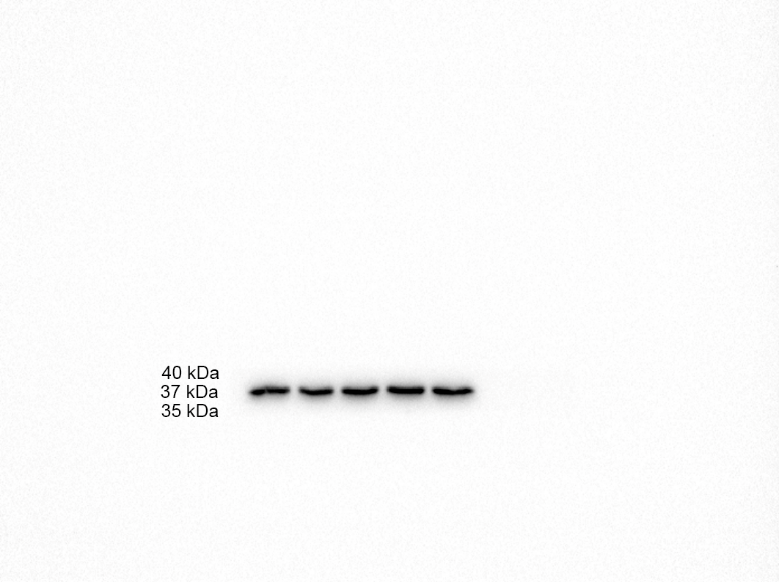


Figure 1H Irg-1 50kDa


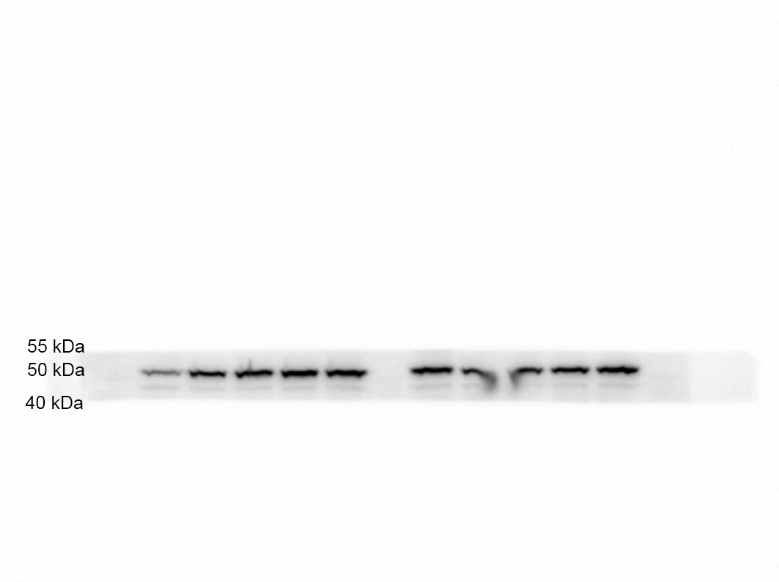


Figure 1H iNOS 131 kDa


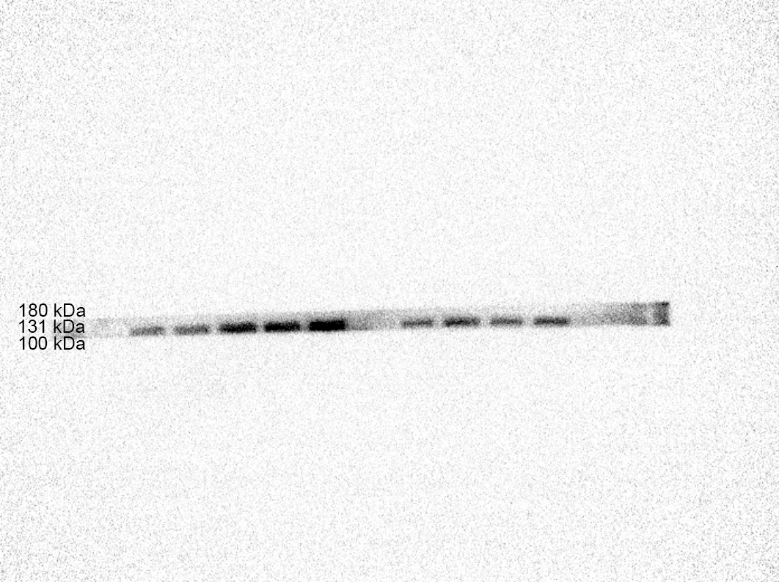


Figure 1H COX2 74 kDa


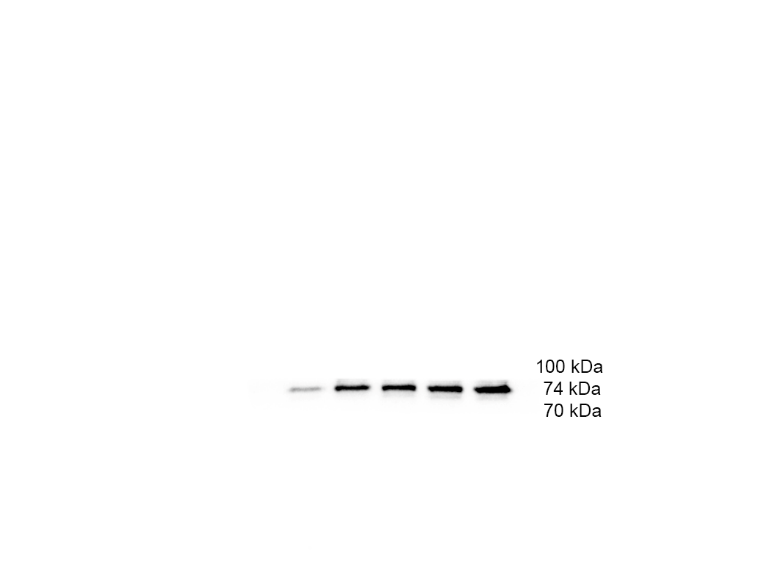


Figure 1H IL-6 24 kDa


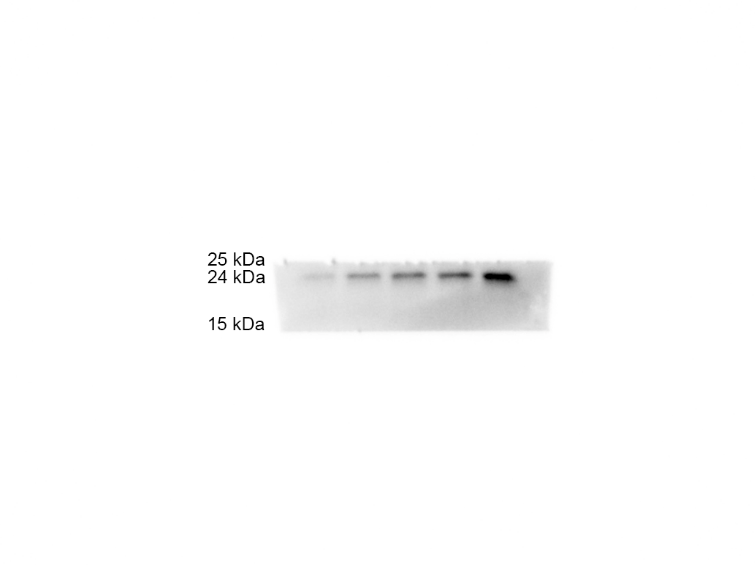


Figure 1H GAPDH 37kDa


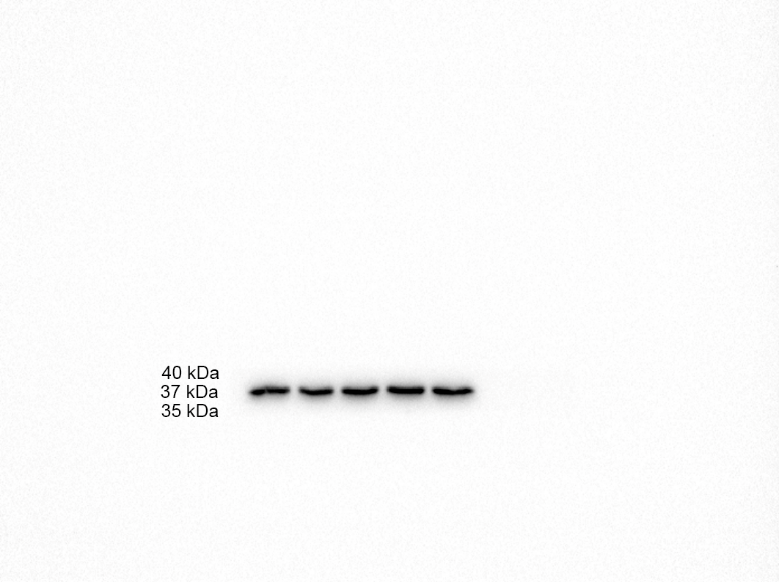


Figure 2A Irg-1 50kDa


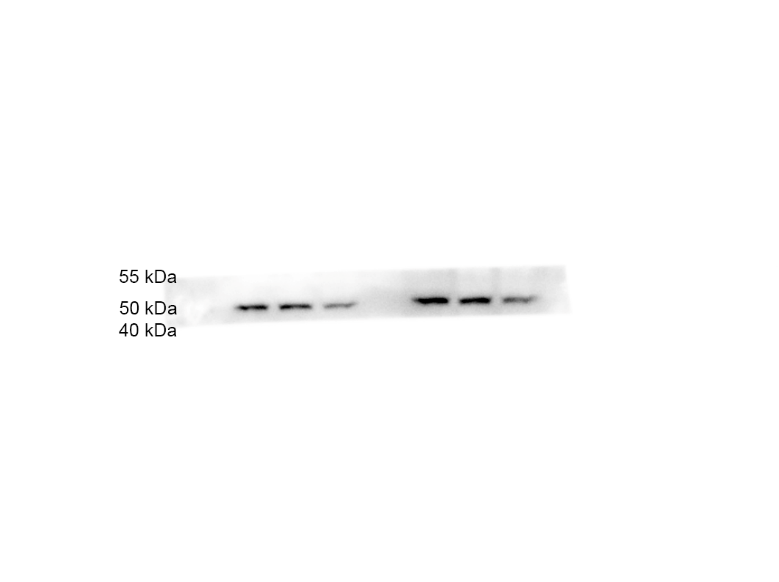


Figure 2A GAPDH 37kDa


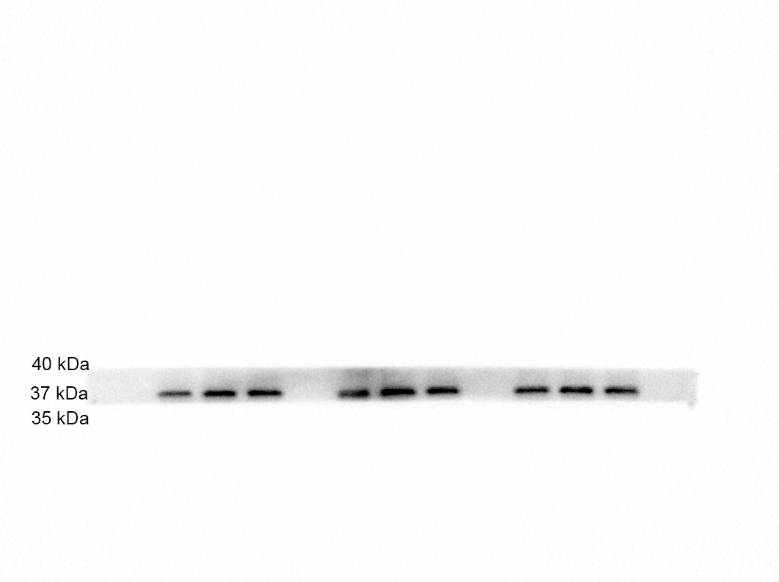


Figure 2D Irg-1 50 kDa


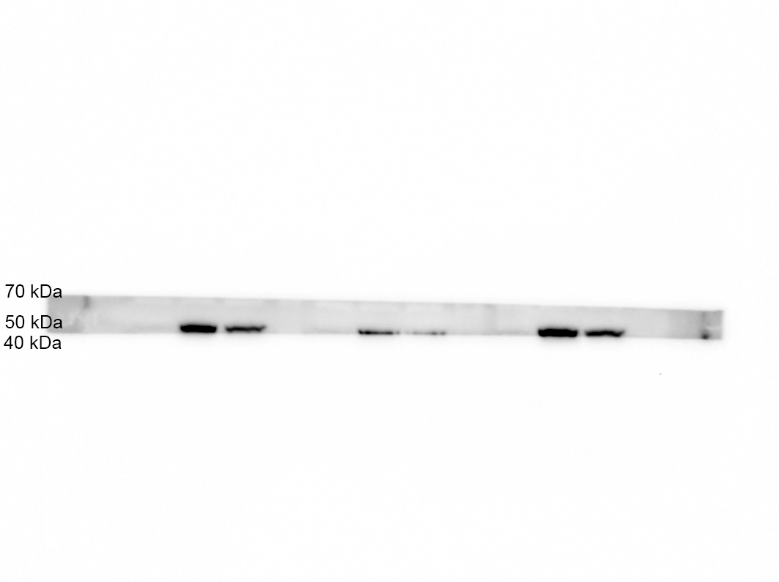


Figure 2D iNOS 131kDa


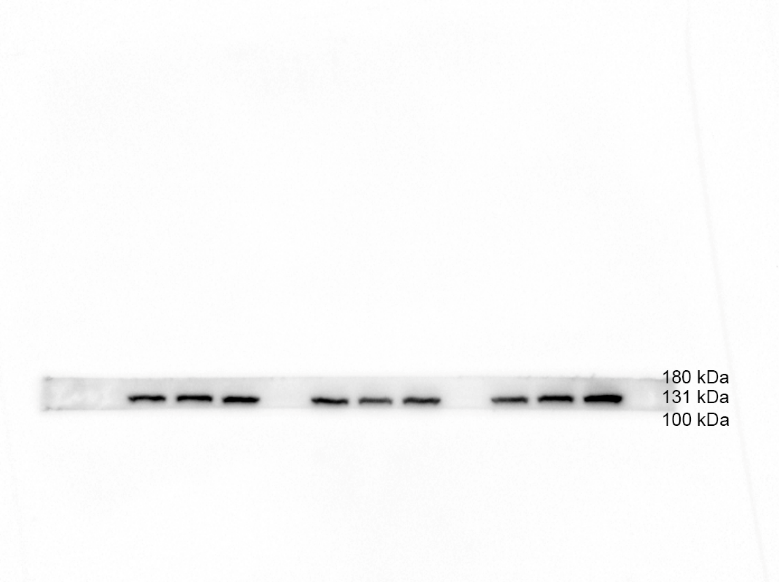


Figure 2D COX2 74kDa


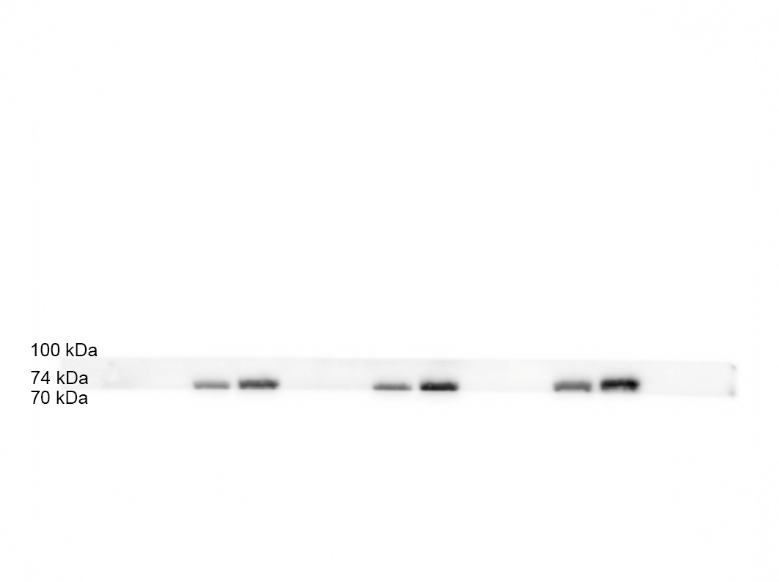


Figure 2D IL-6 24kDa


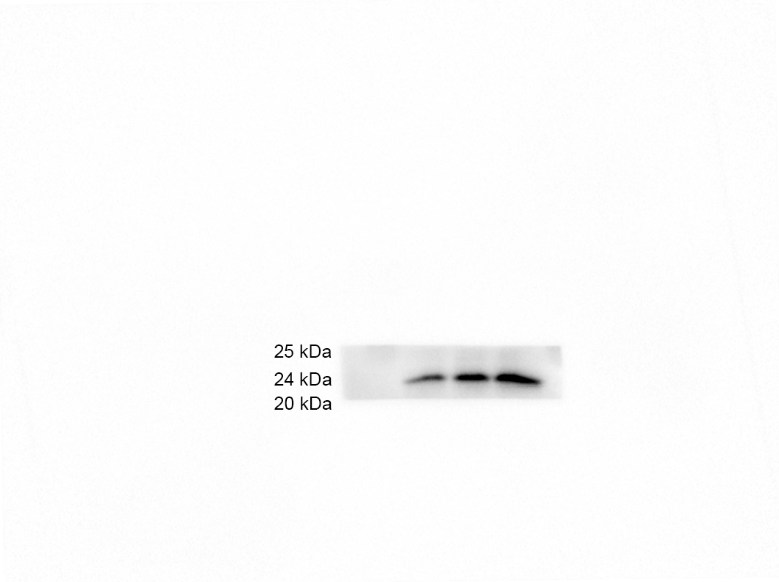


Figure 2D GAPDH 37kDa


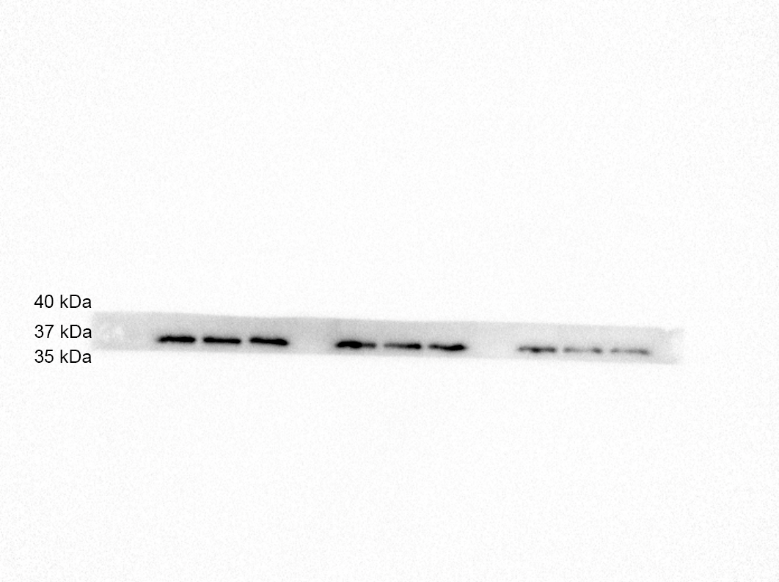


Figure 2G Irg-1 50kDa


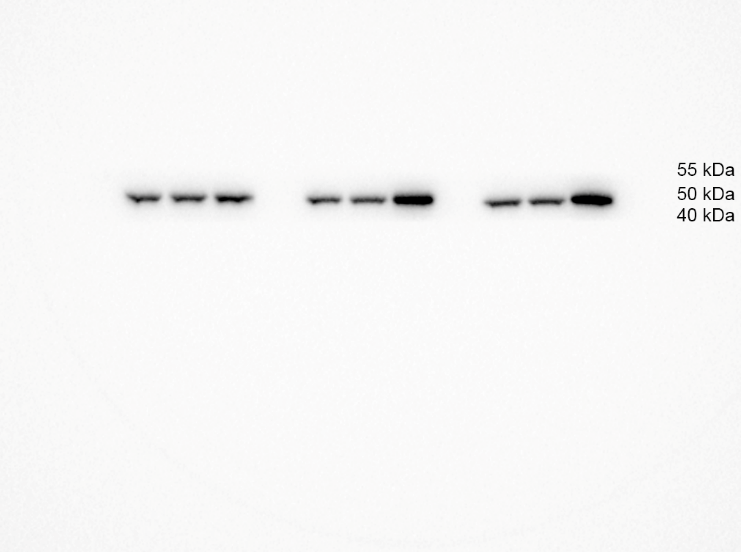


Figure 2G GAPDH 37kDa


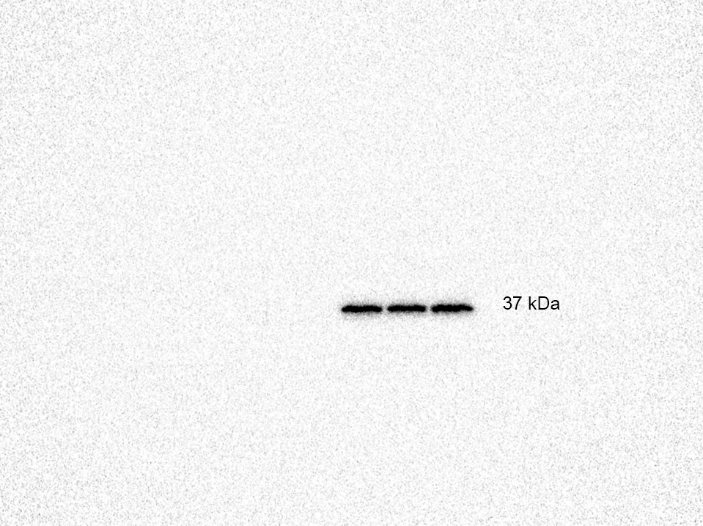


Figure 2J Irg-1 50 kDa


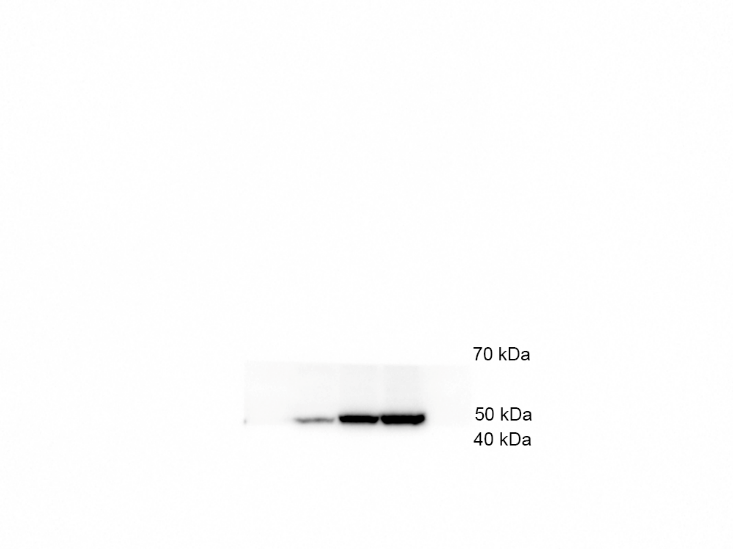


Figure 2J iNOS 131kDa


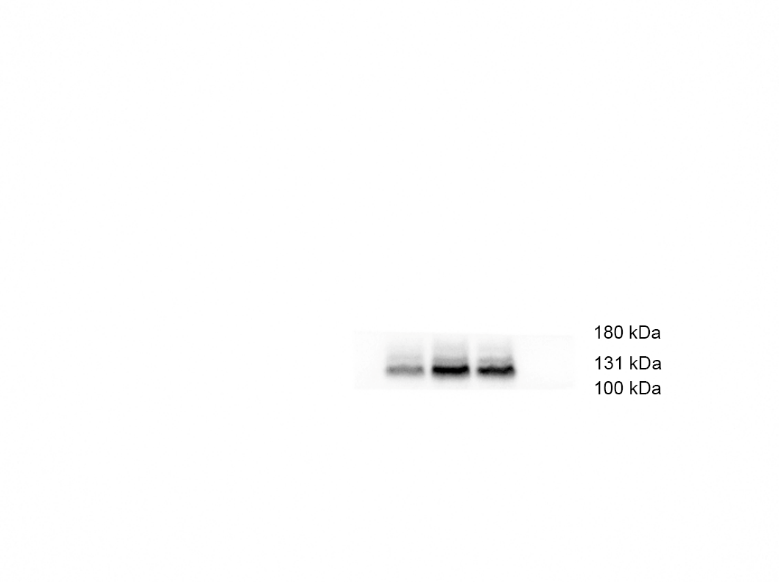


Figure 2J COX2 74kDa


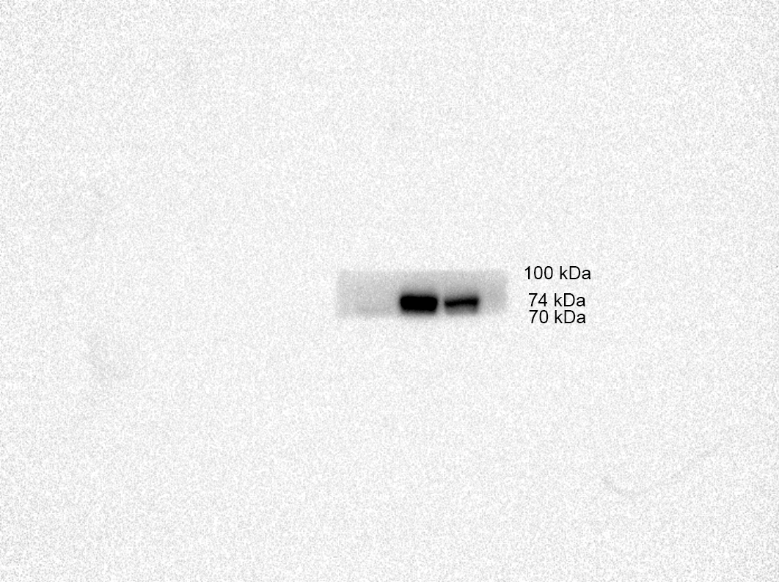


Figure 2J IL-6 24kDa


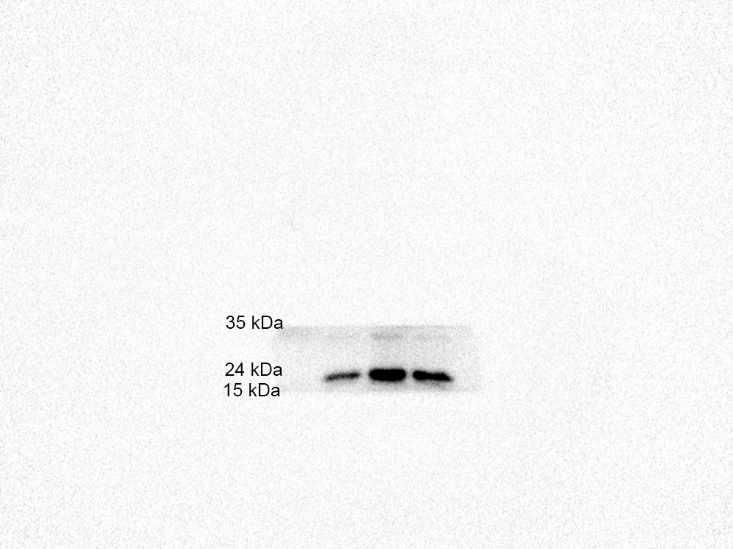


Figure 2J GAPDH 37kDa


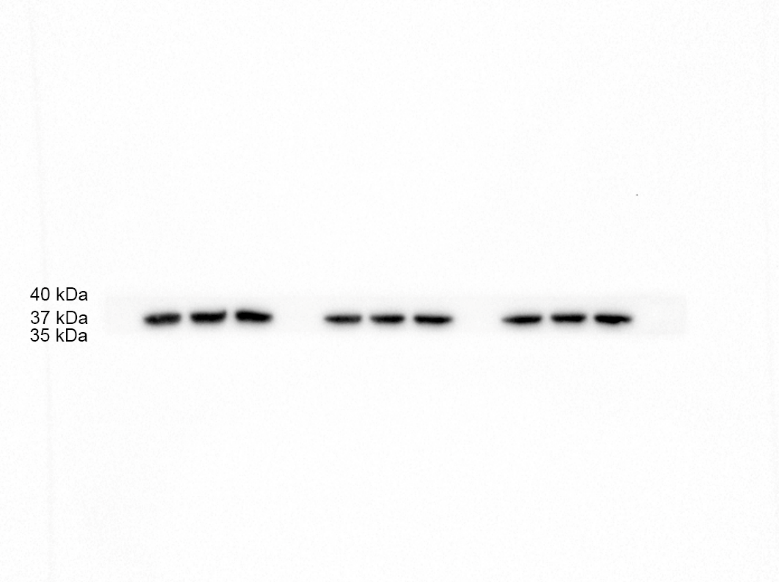


Figure 3A Nrf2 110 kDa


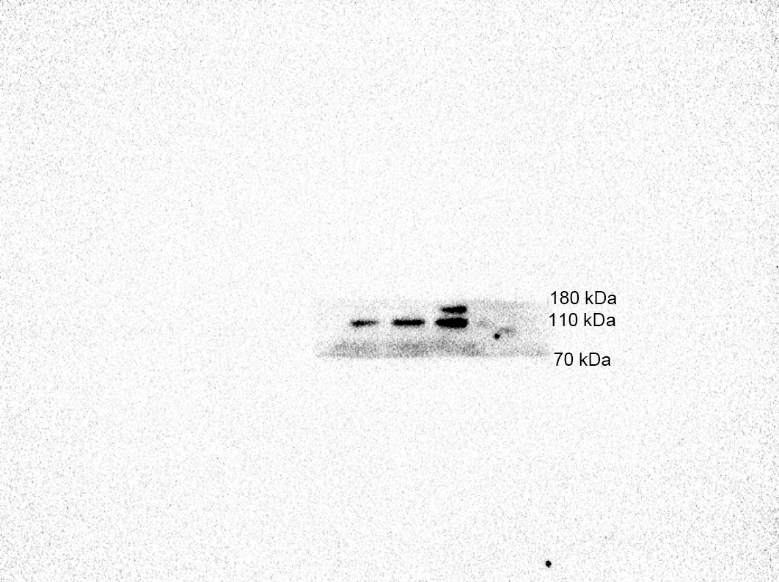


Figure 3A Lamin B 66kDa


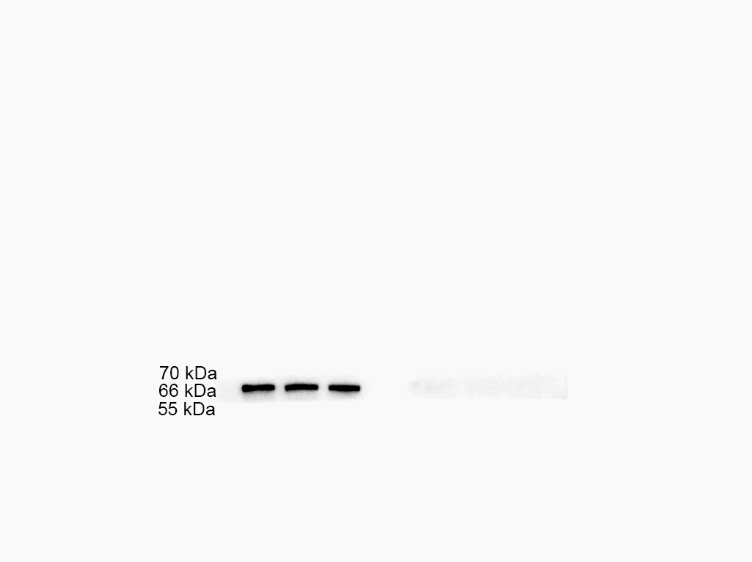


Figure 3A HO-1 33kDa


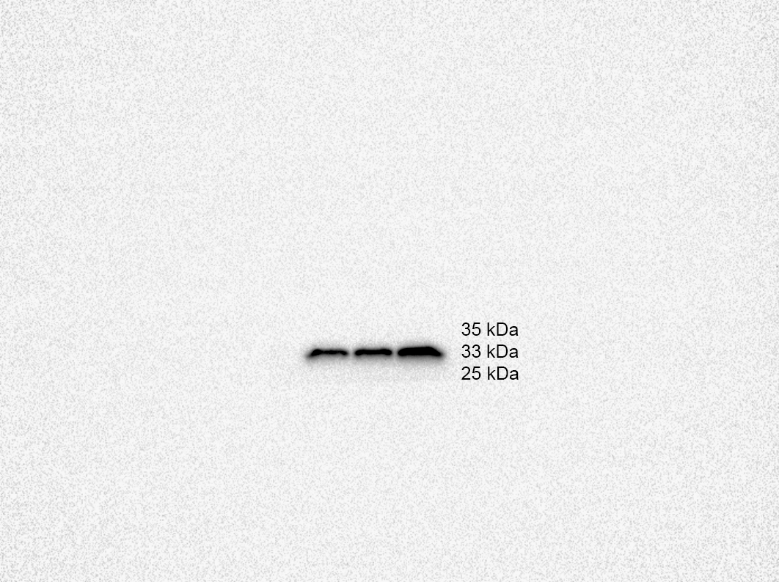


Figure 3A NQO1 31kDa


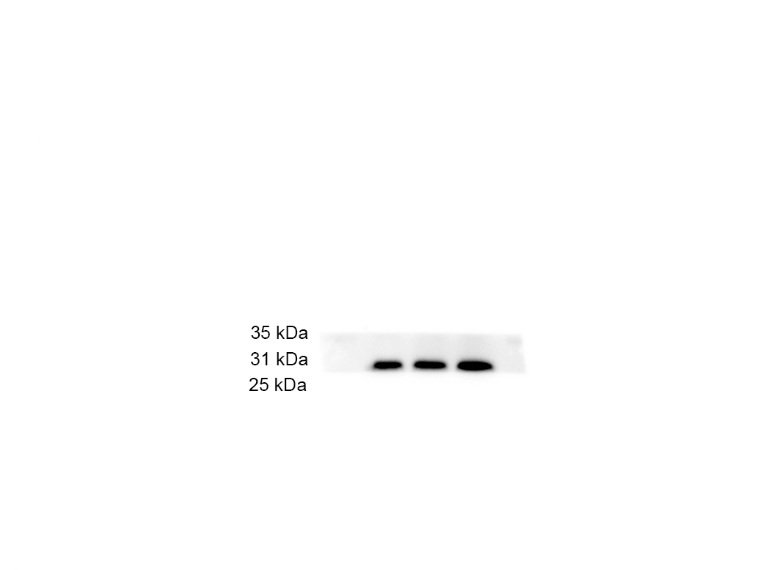


Figure 3A GAPDH 37kDa


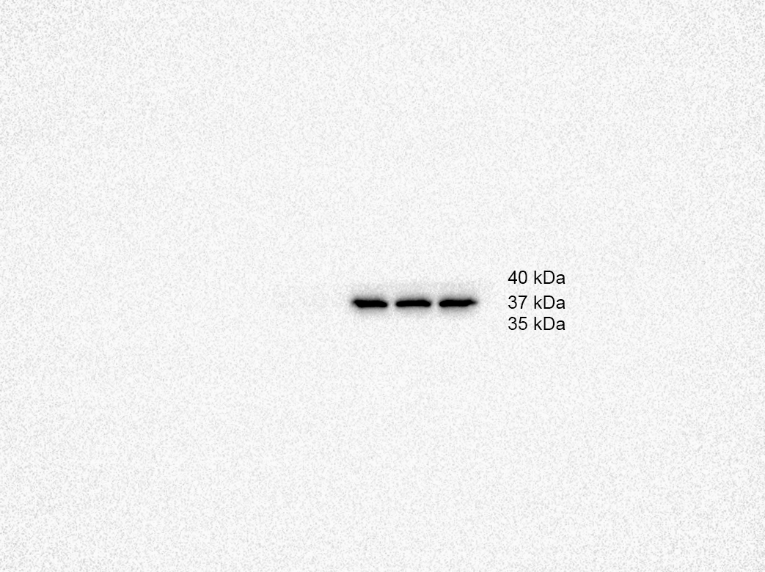


Figure 3D Nrf2 110kDa


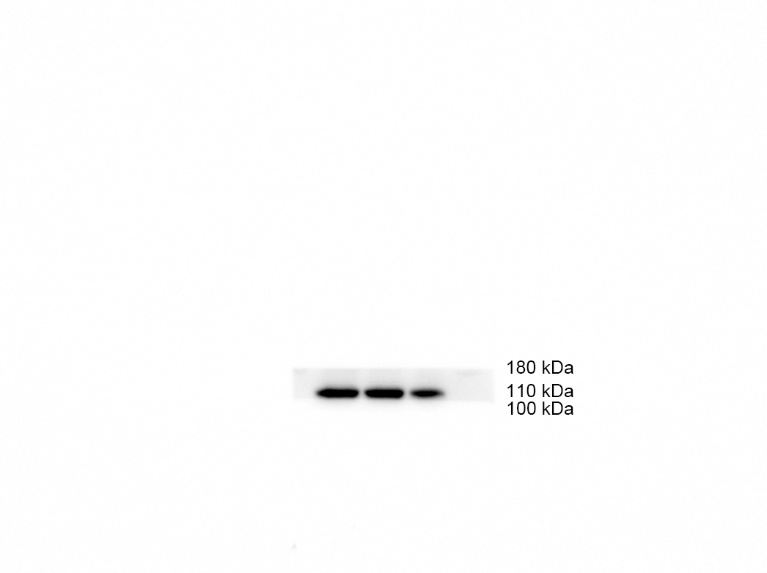


Figure 3D GAPDH 37kDa


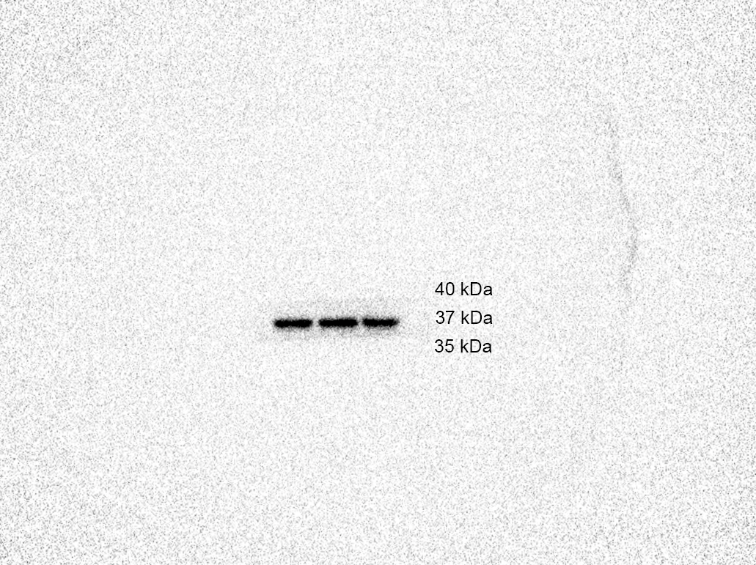


Figure 3F Nrf2 110 kDa


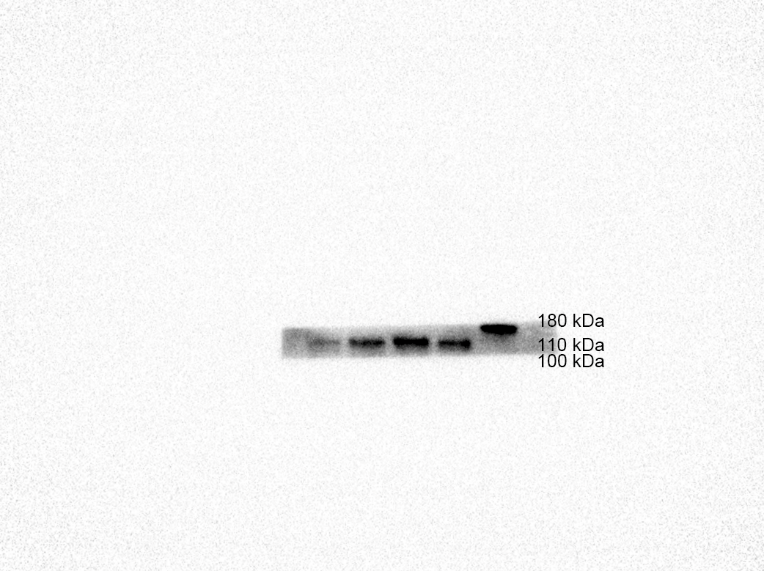


Figure 3F Lamin B 66kDa


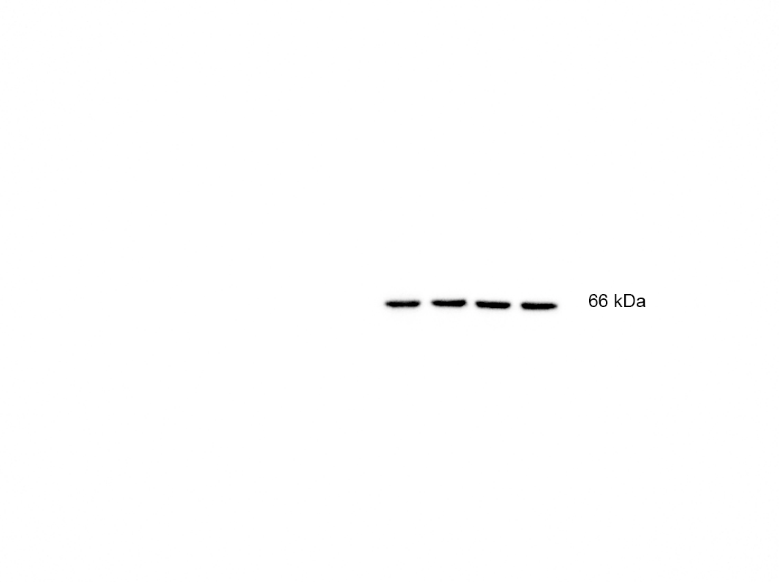


Figure 3F HO-1 33kDa


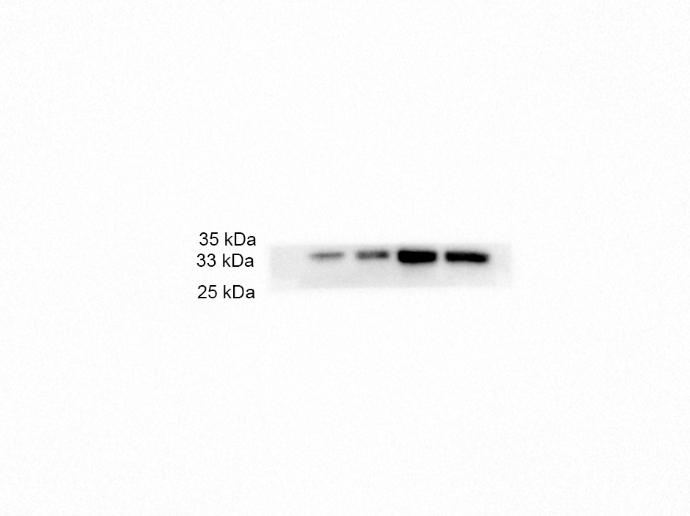


Figure 3F NQO1 31kDa


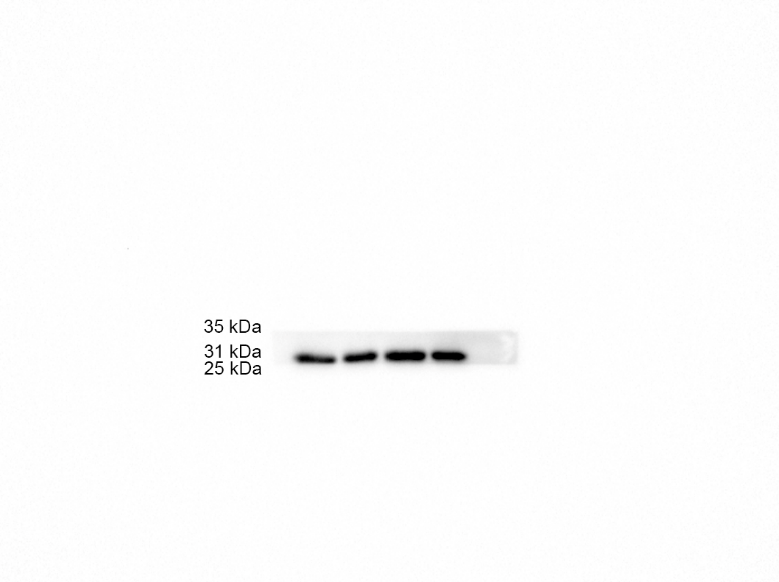


Figure 3F GAPDH 37kDa


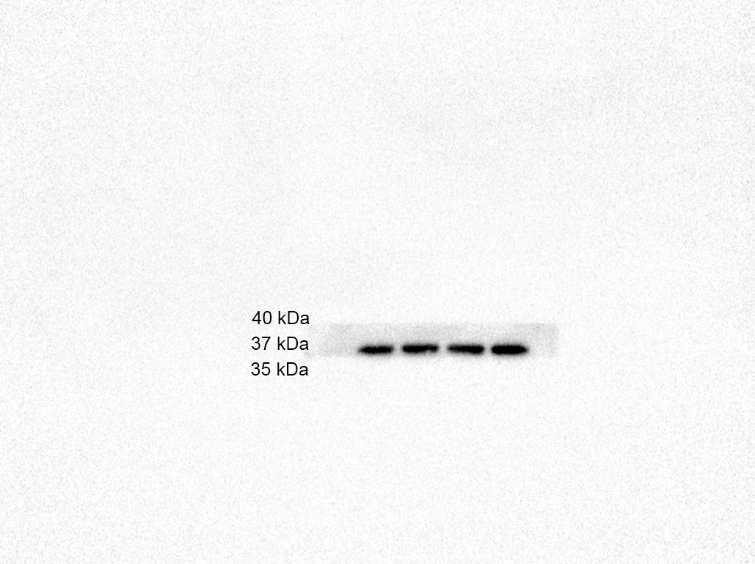


Figure 3J iNOS 131kDa


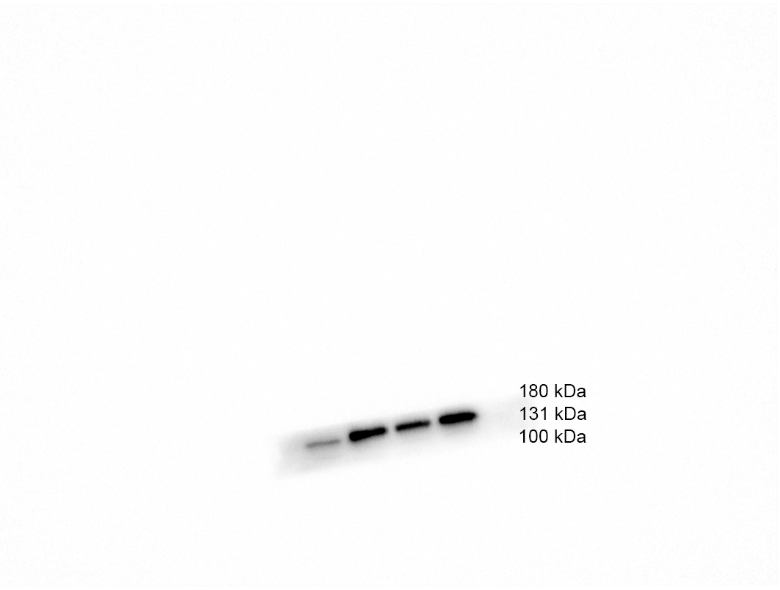


Figure 3J COX2 74kDa


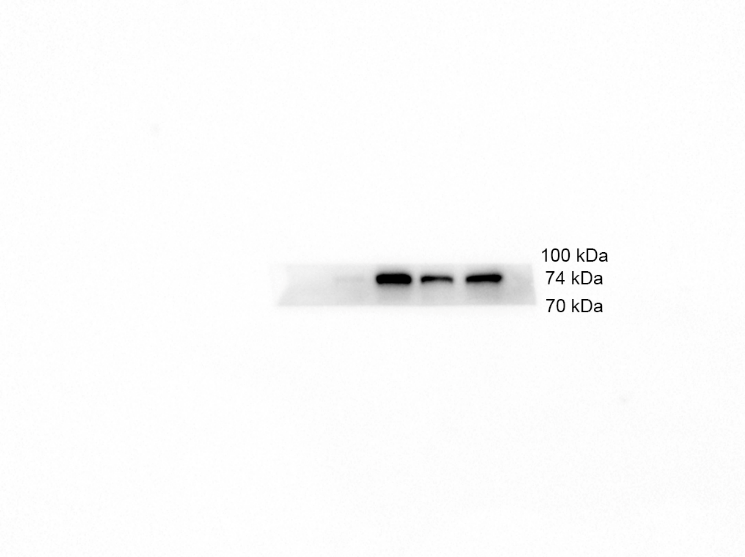


Figure 3J IL-6 24kDa


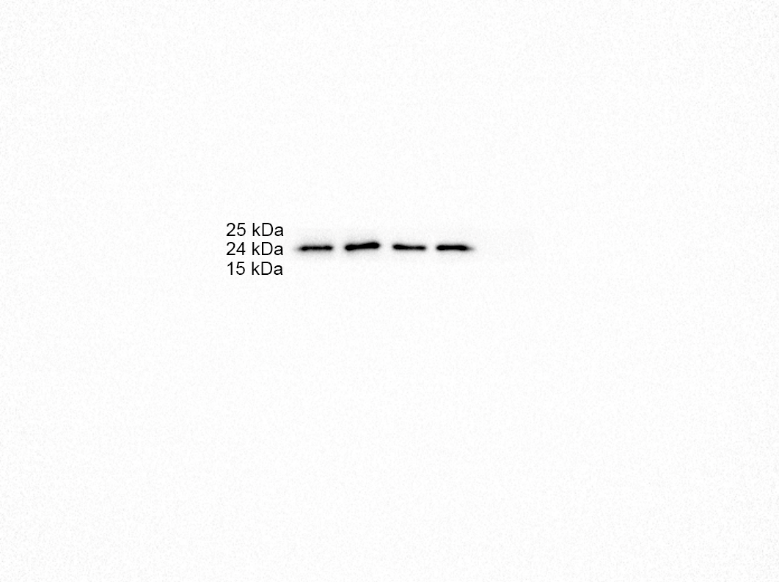


Figure 3J GAPDH 37kDa


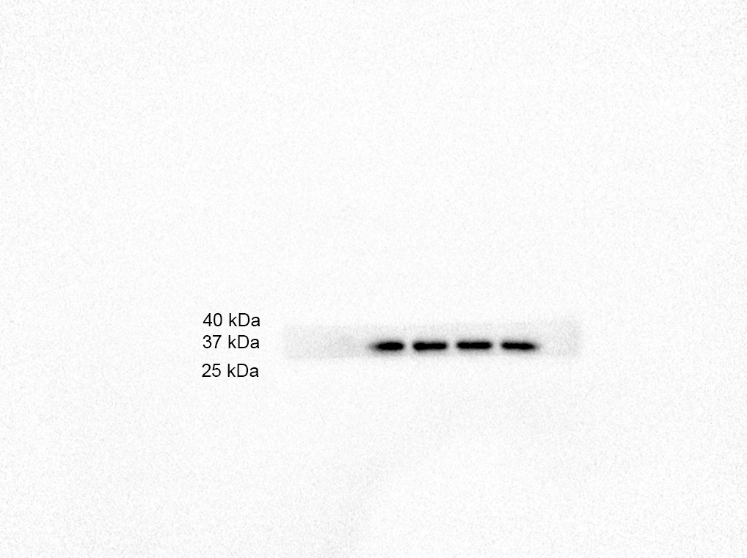


Figure 4A Irg-1 50kDa


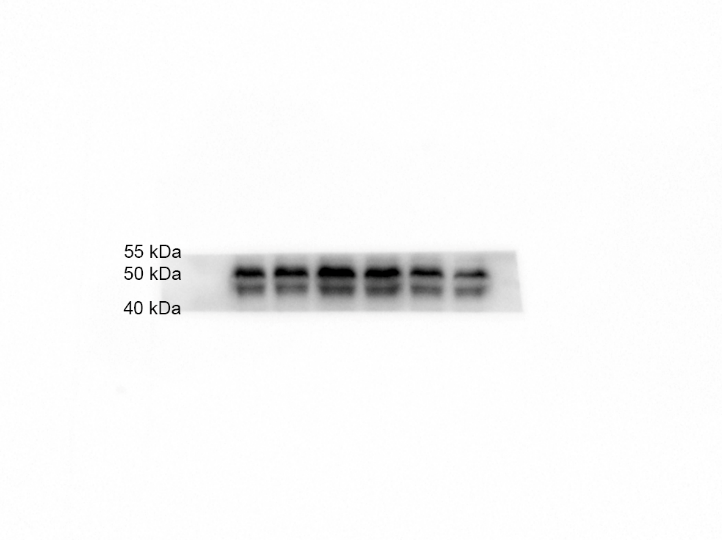


Figure 4A GAPDH 37kDa


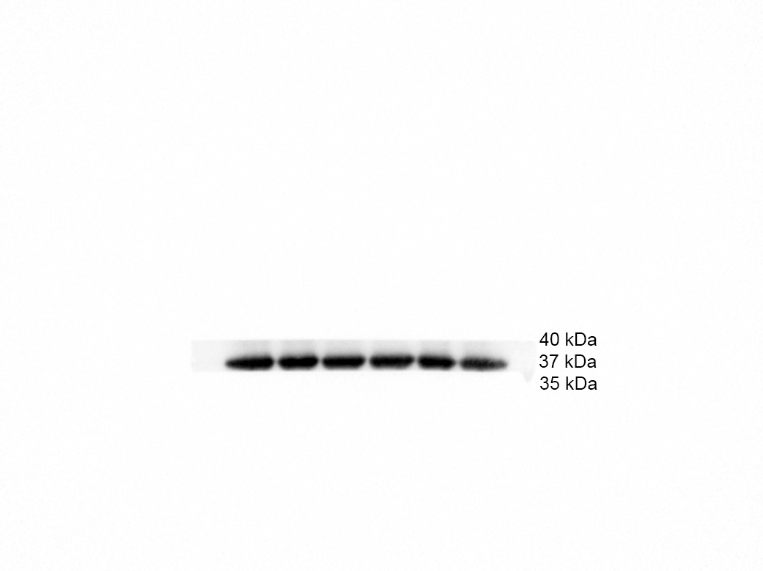


Figure 5G iNOS 131kDa


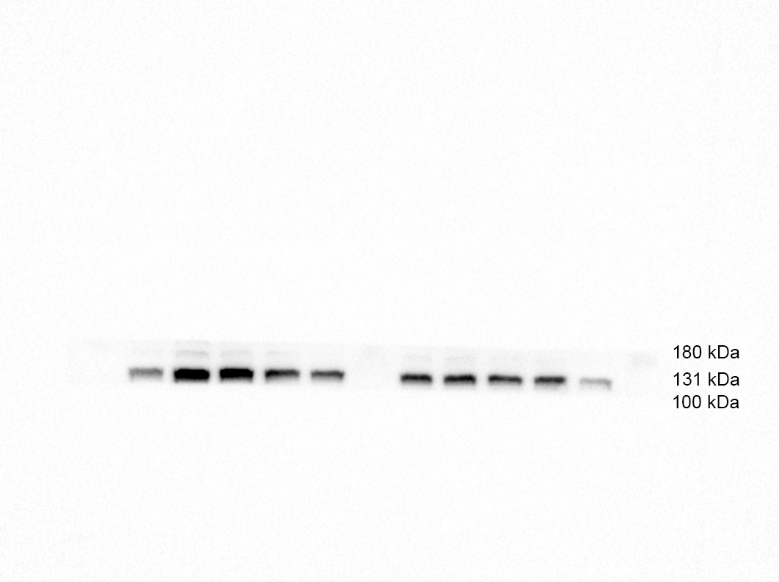


Figure 5G COX2 74kDa


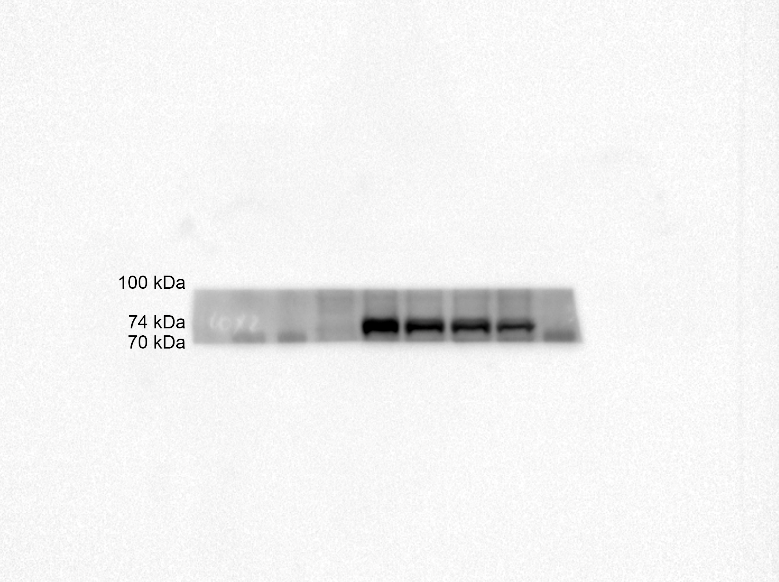


Figure 5G IL-6 24kDa


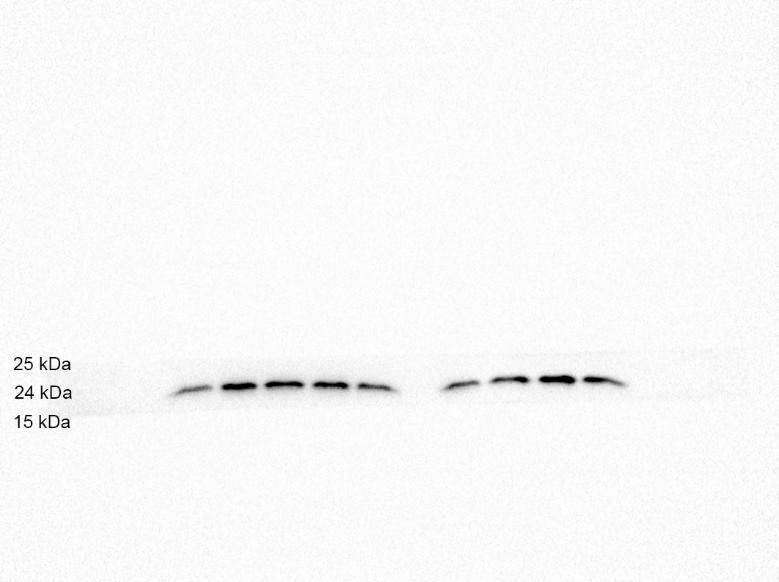


Figure 5G GAPDH 37kDa


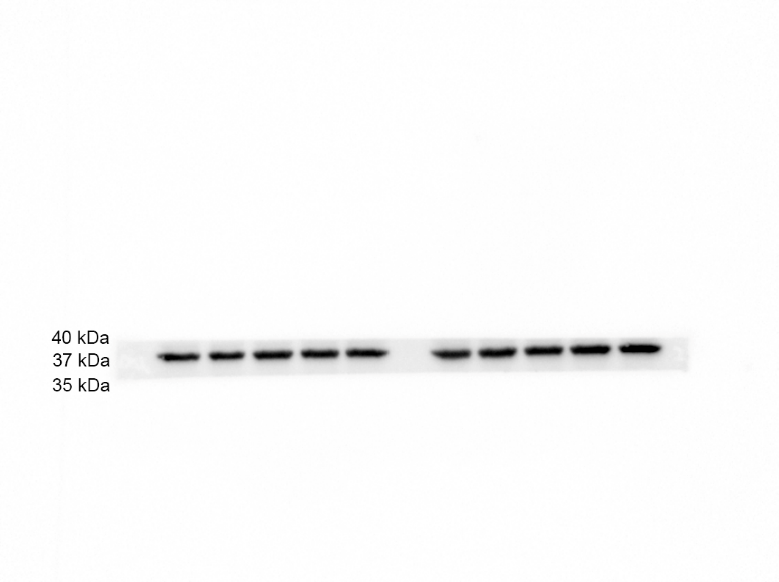


Figure 6D Iba-1


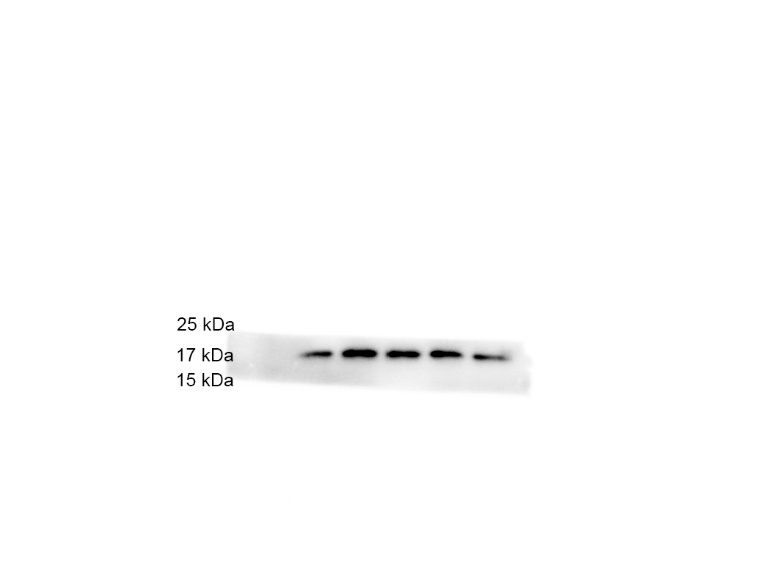


Figure 6D CD68 100kDa


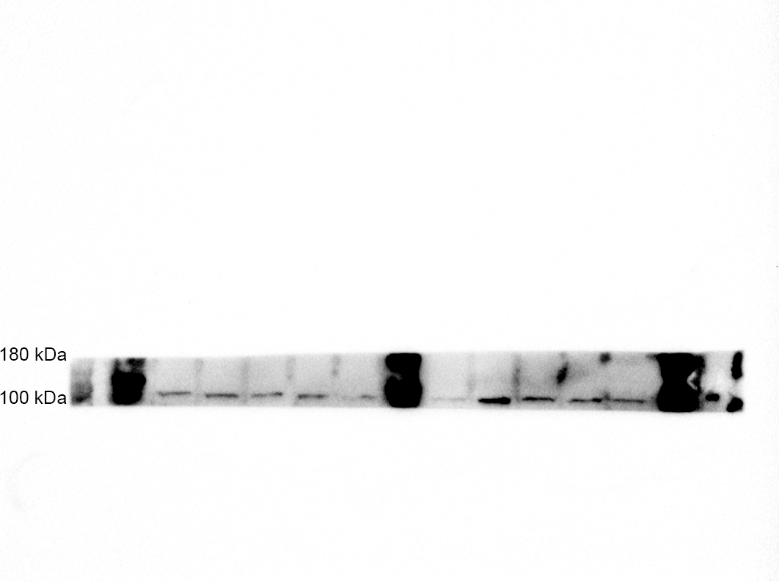


Figure 6D Arg-1 36kDa


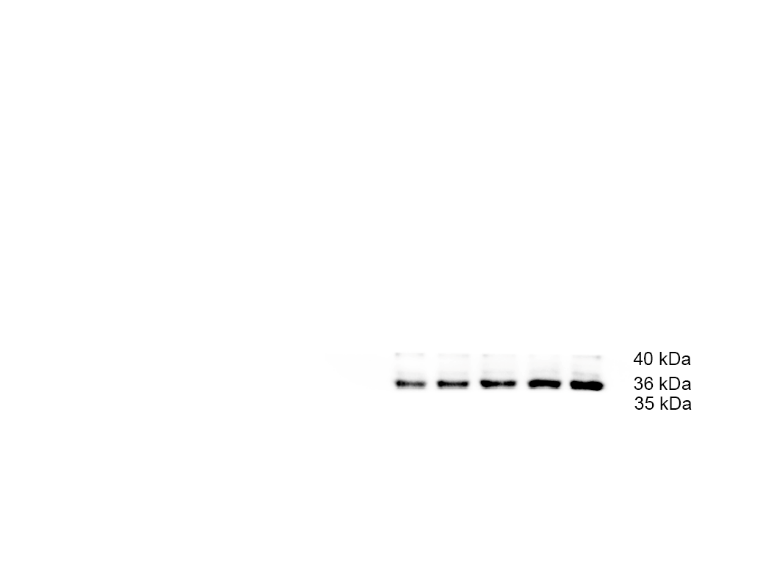


Figure 6D GAPDH 37kDa


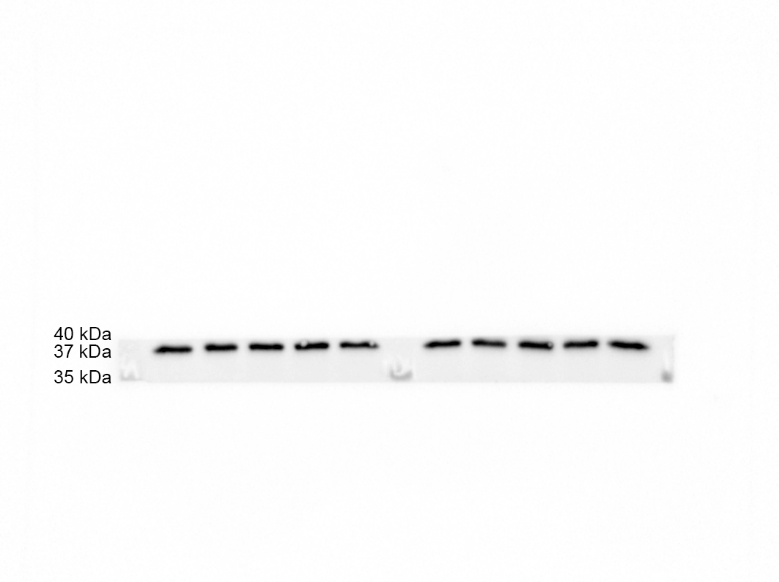


Figure 7A Bcl2 26kDa


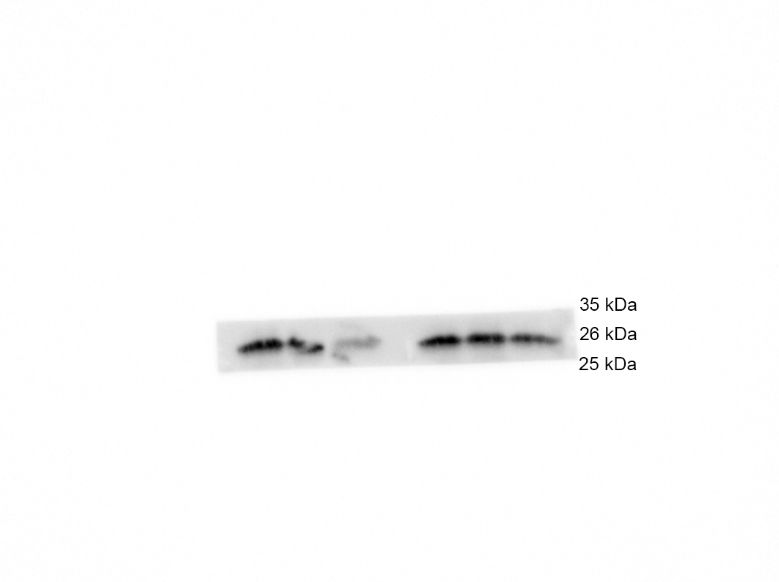


Figure 7A Bax 21kDa


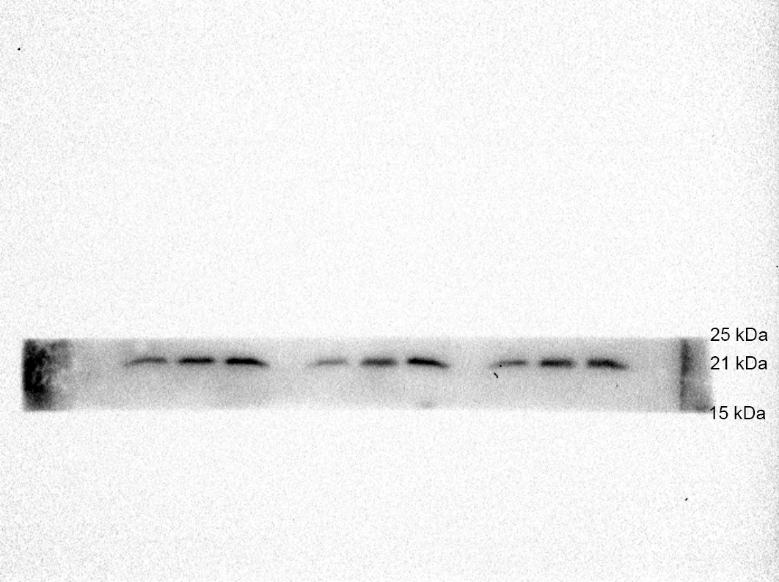


Figure 7A C-caspase3 17kDa


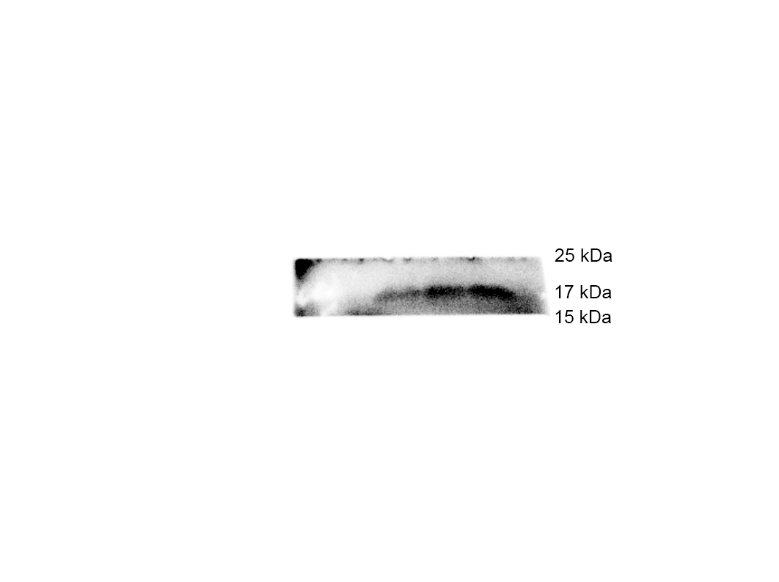


Figure 7A GADPH 37kDa


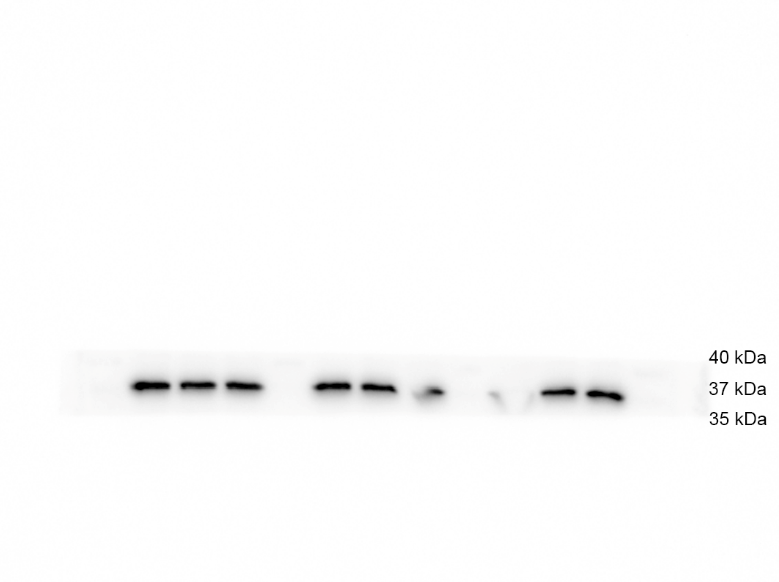


Figure 7C Bcl2 26kDa


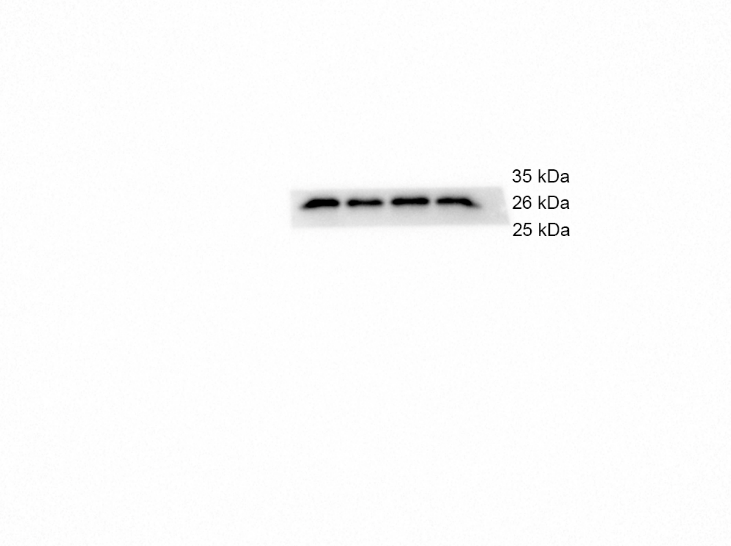


Figure 7C Bax 21kDa


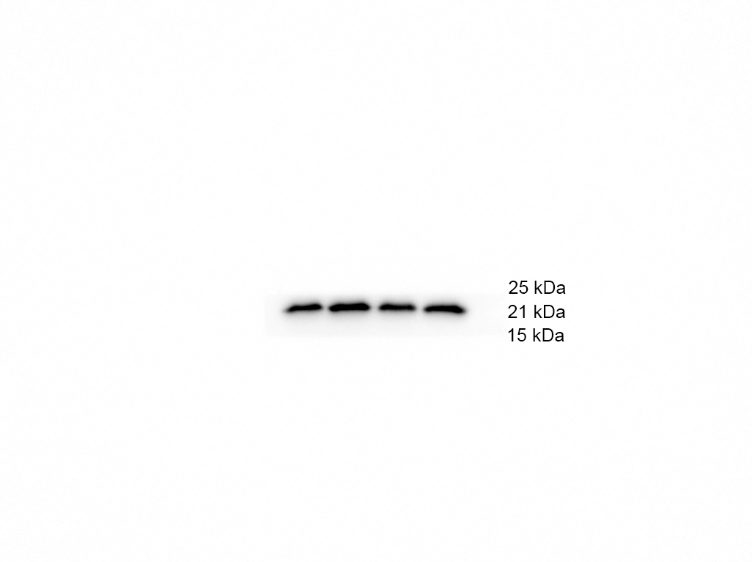


Figure 7C C-caspase3 17kDa


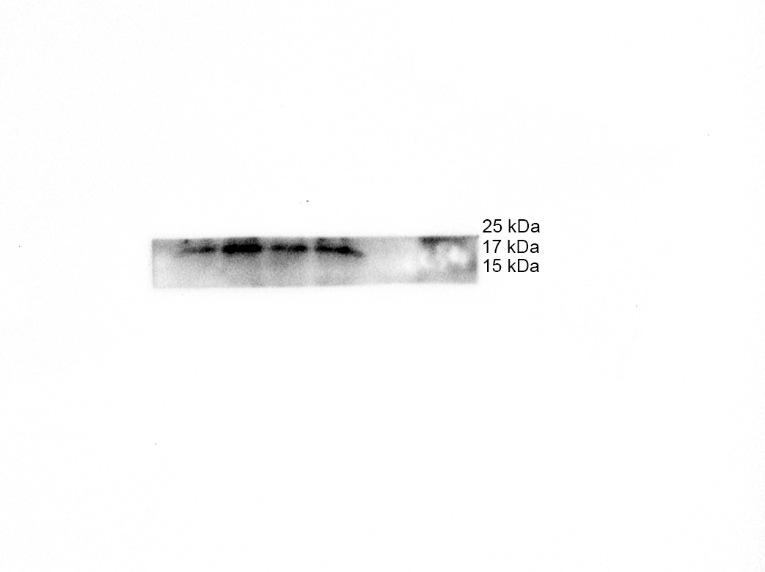


Figure 7C GAPDH 37kDa


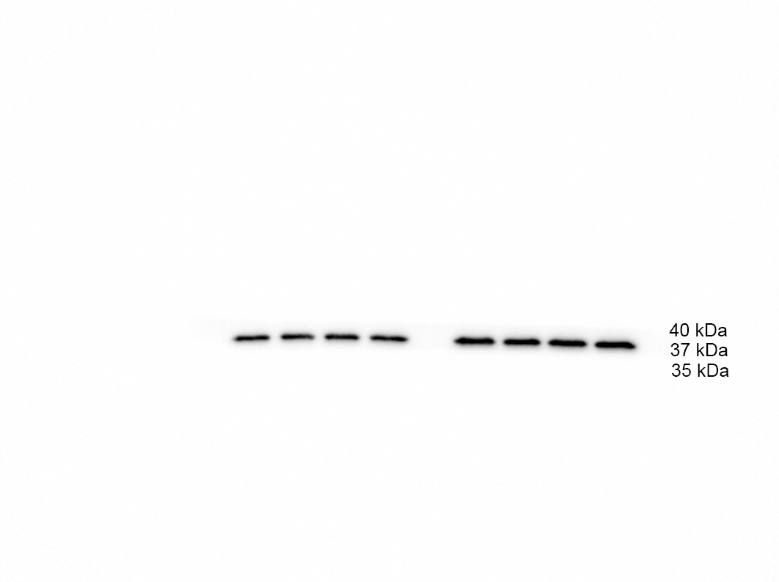


Figure 8A Nrf2 110kDa


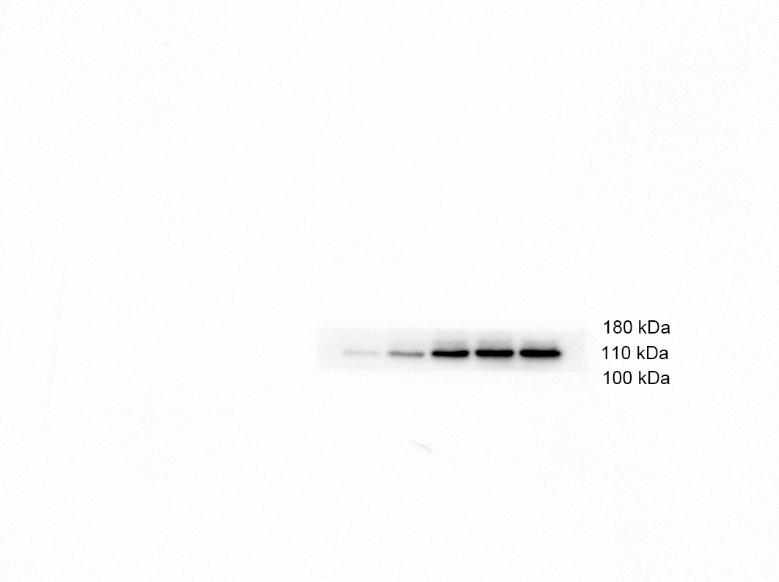


Figure 8A Lamin B 66kDa


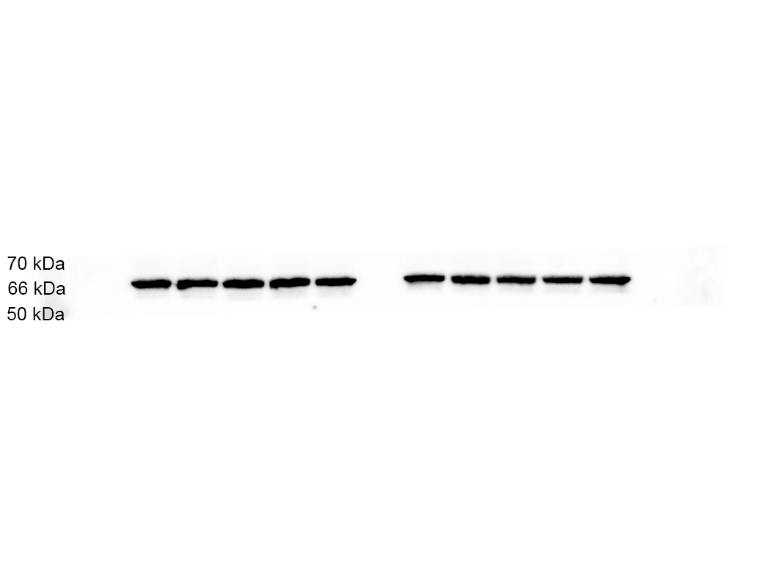


Figure 8A HO-1 33kDa


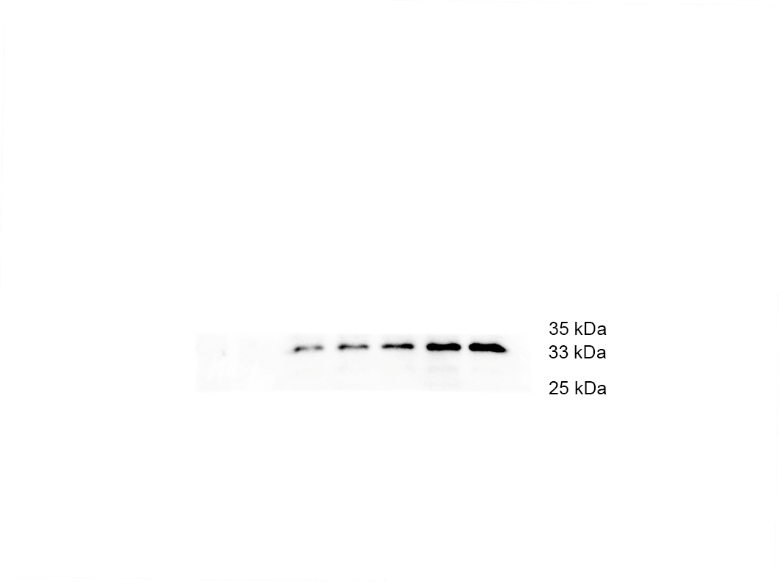


Figure 8A NQO1 31kDa


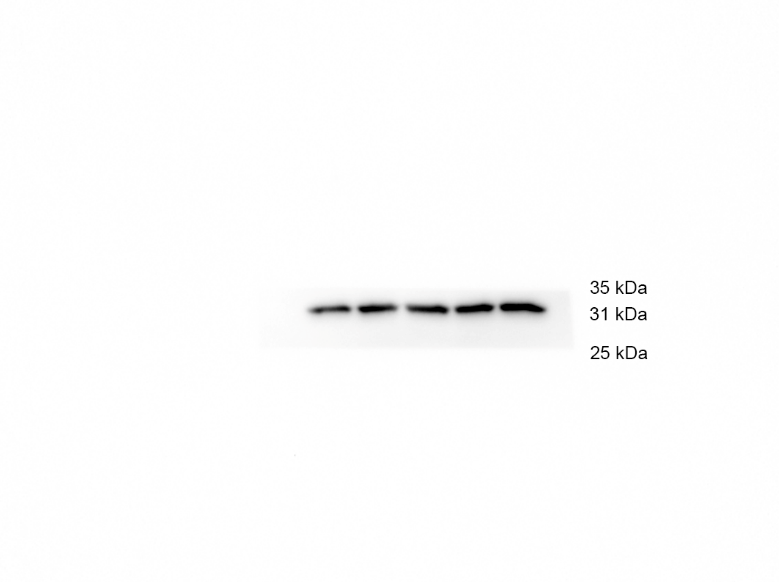


Figure 8A GADPH 37kDa


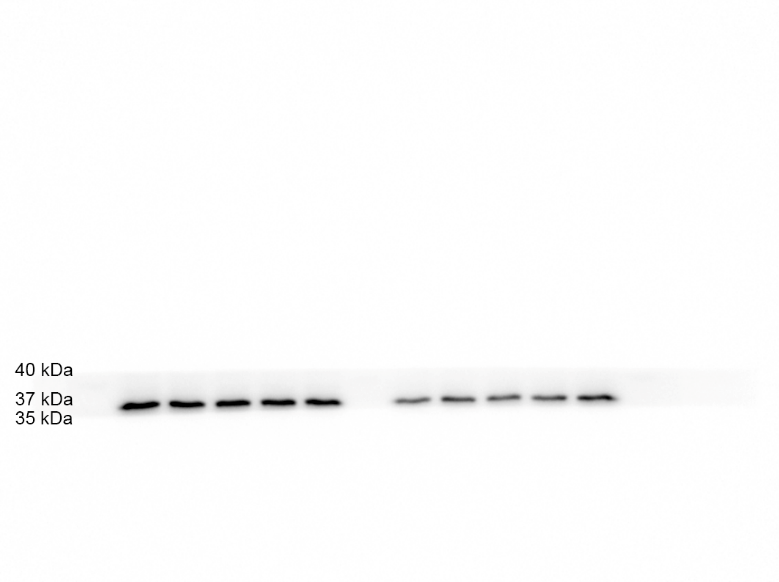


Figure 8F Nrf2 110kDa


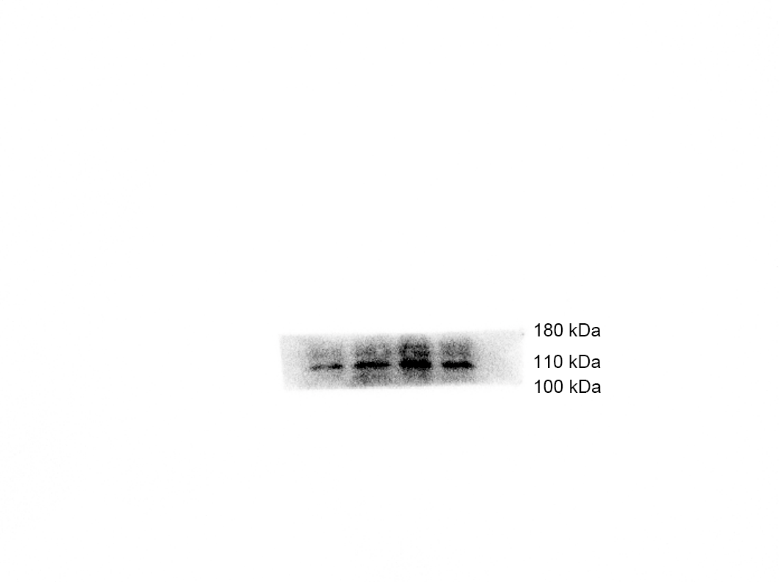


Figure 8F Lamin B 66kDa


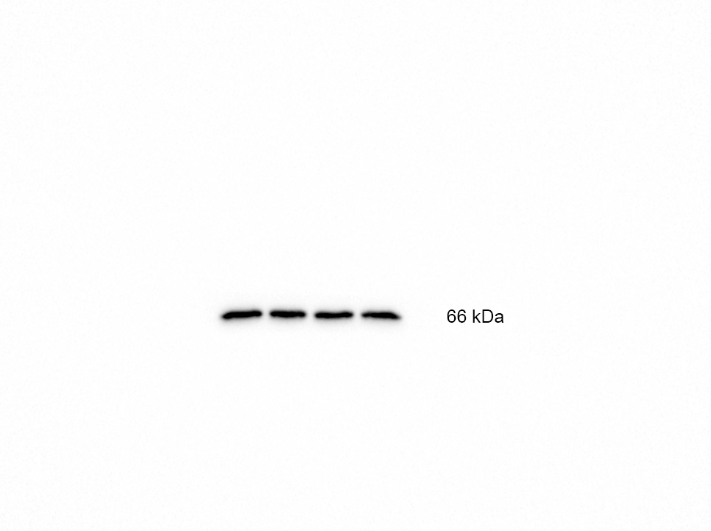


Figure 8F HO-1 33kDa


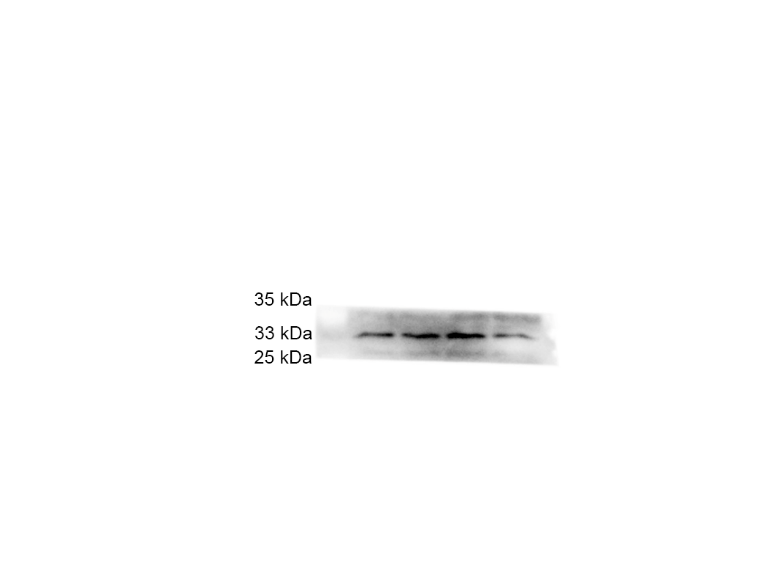


Figure 8F NQO1 31kDa


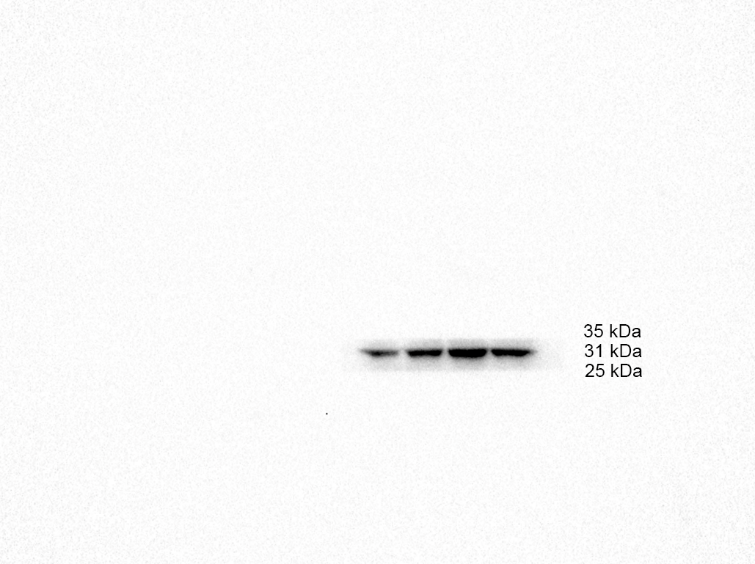


Figure 8F GAPDH 37kDa


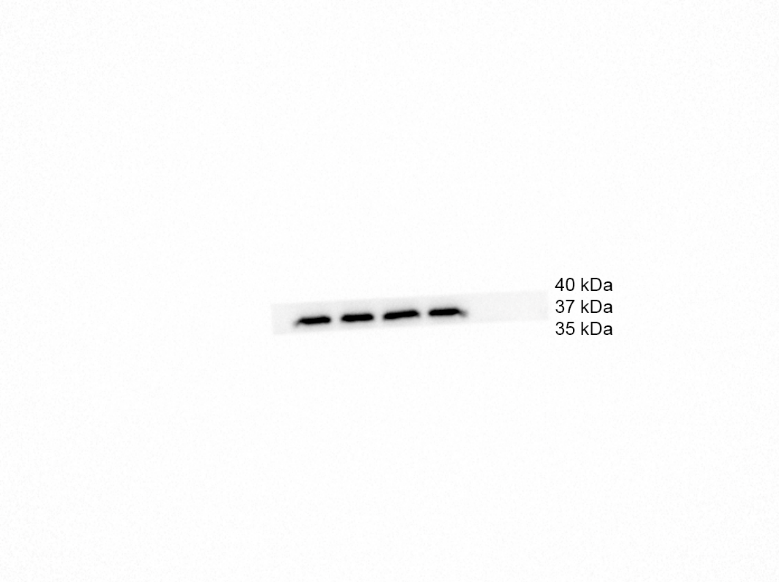


Figure 8H iNOS 131kDa


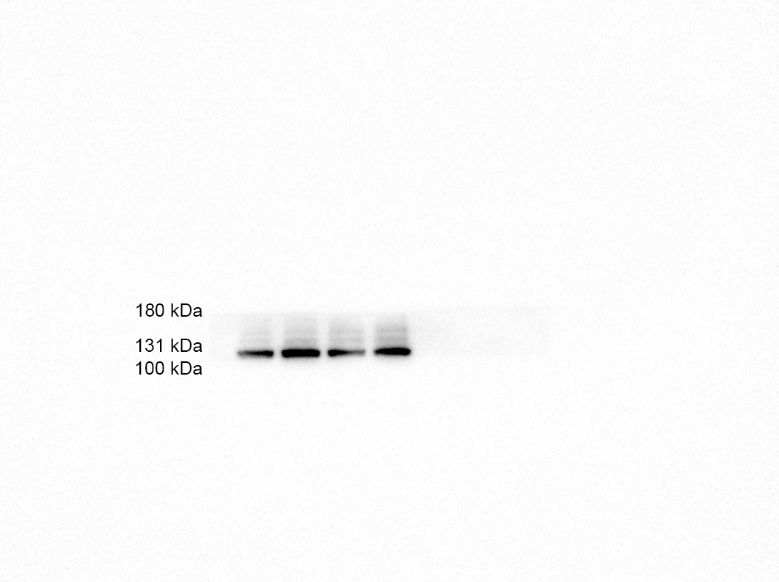


Figure 8H COX2 74 kDa


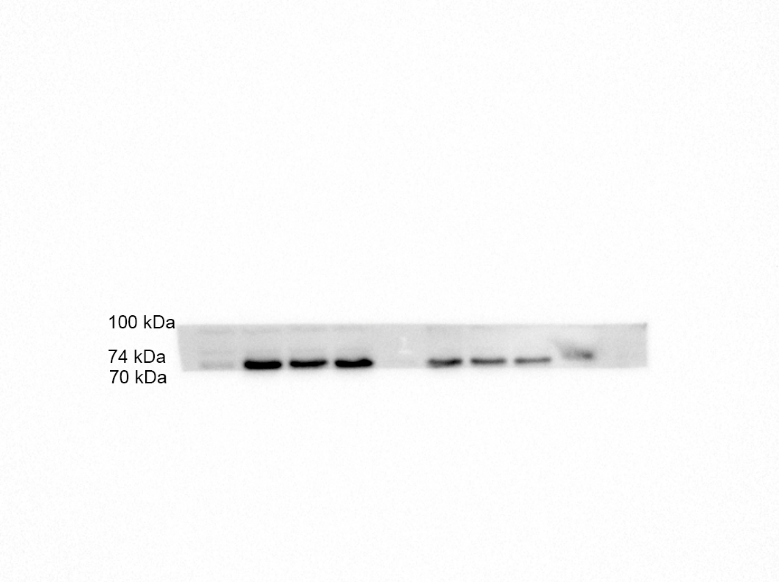


Figure 8H IL-6 24kDa


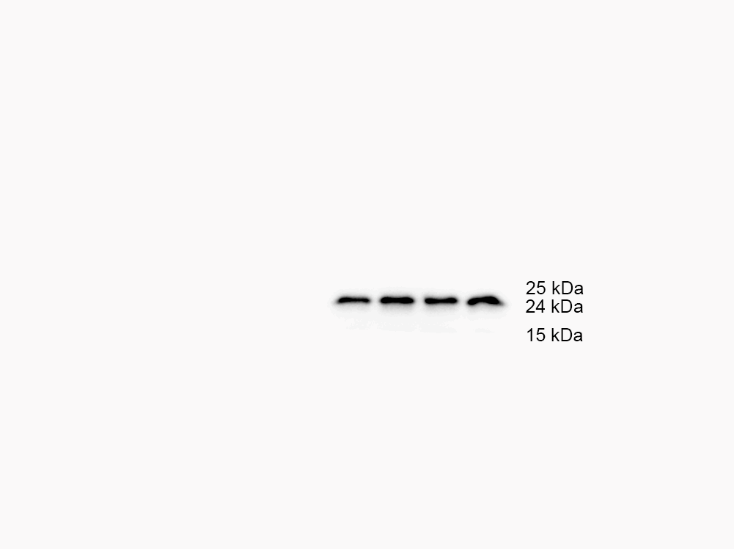


Figure 8H GADPH 37kDa


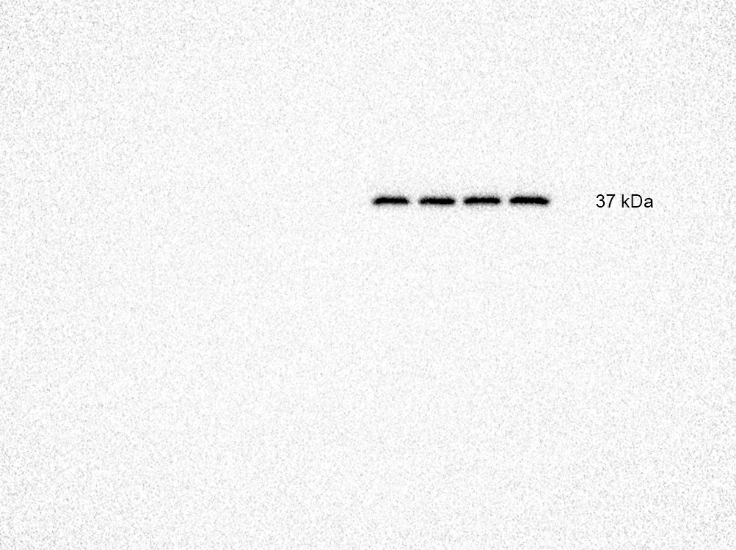


Figure 9F iNOS 131kDa


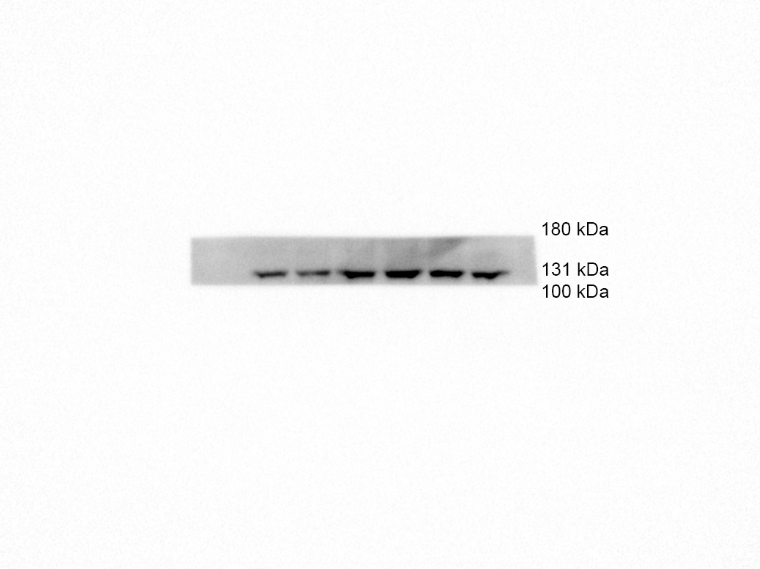


Figure 9F COX2 74kDa


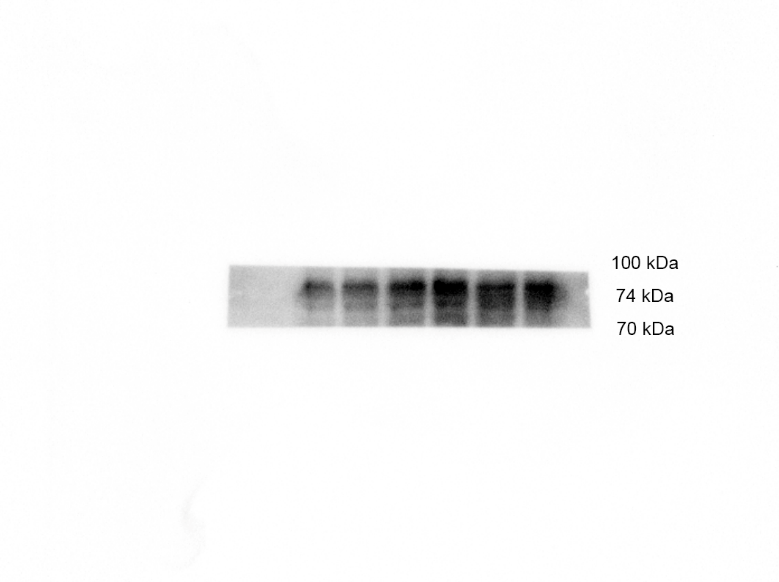


Figure 9F GAPDH 37kDa


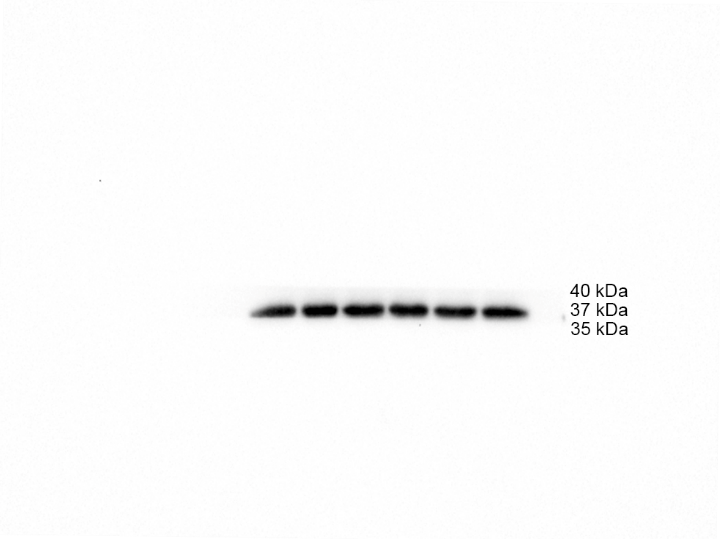


Figure S3A Nrf2 110kDa


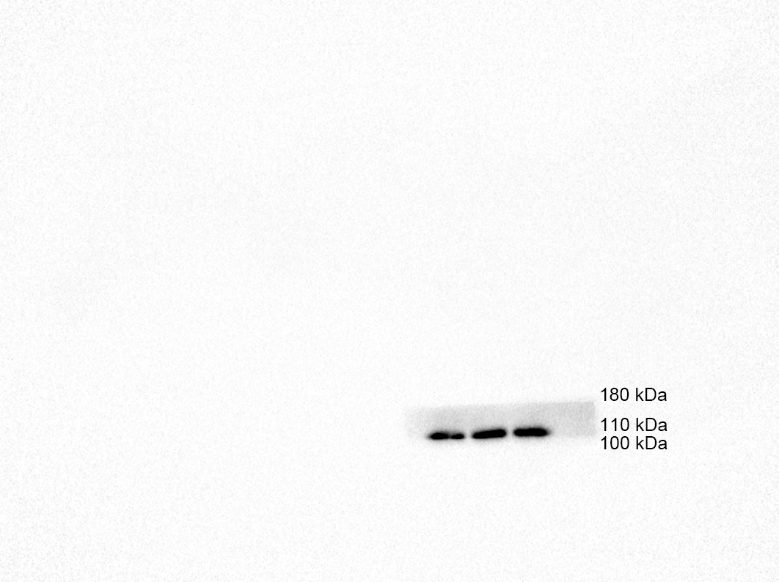


Figure S3A GAPDH 37kDa


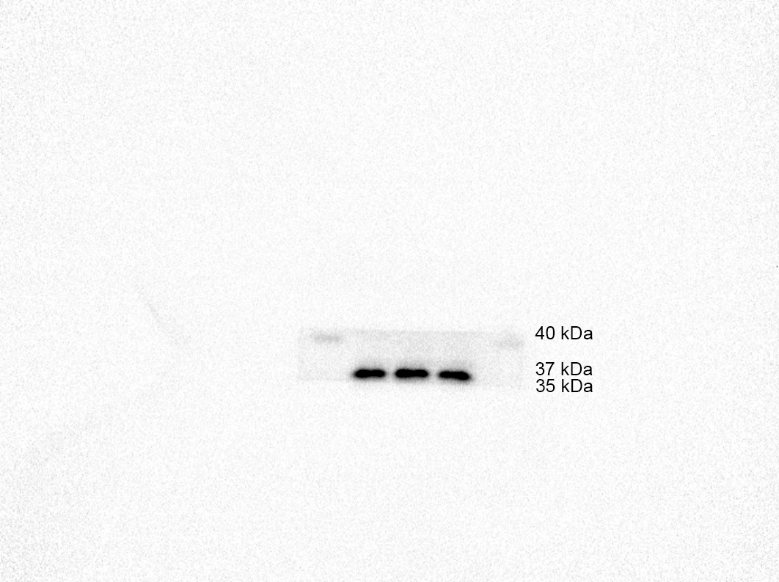


Figure S3A Nrf2 110kDa


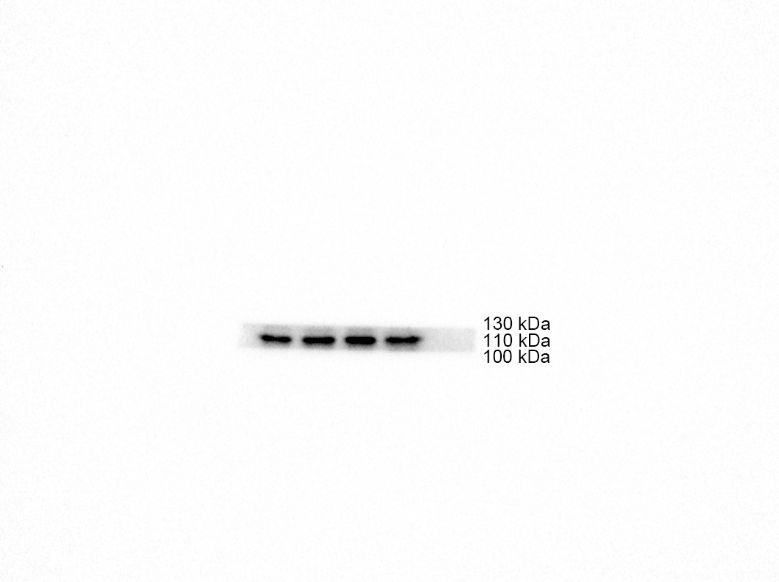


Figure S3C GAPDH 37kDa


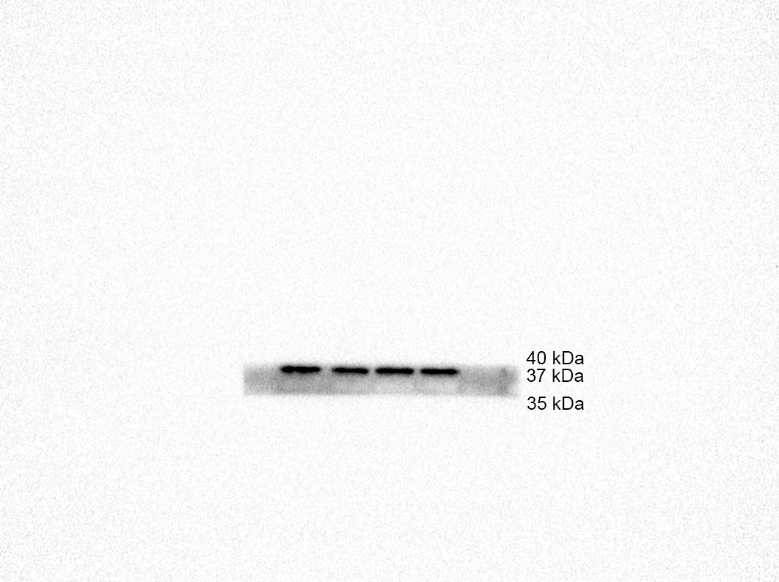


Figure S3E Nrf2 110kDa


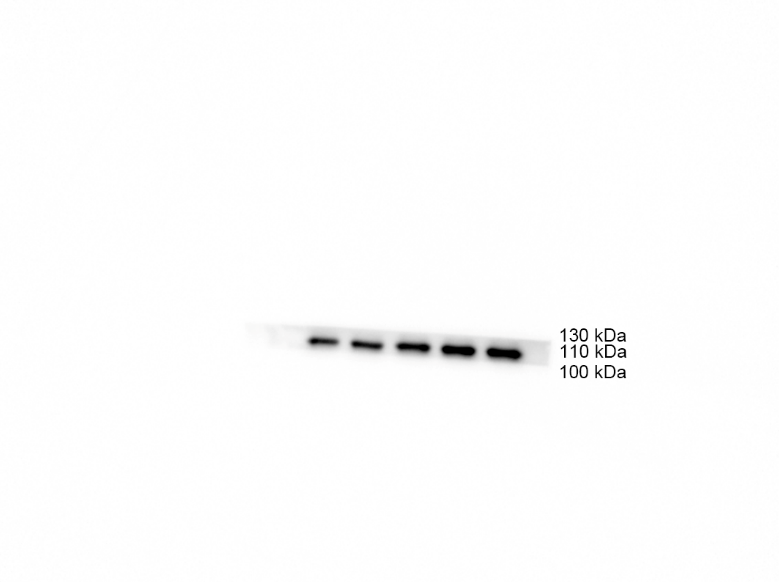


Figure S3E GAPDH 37kDa


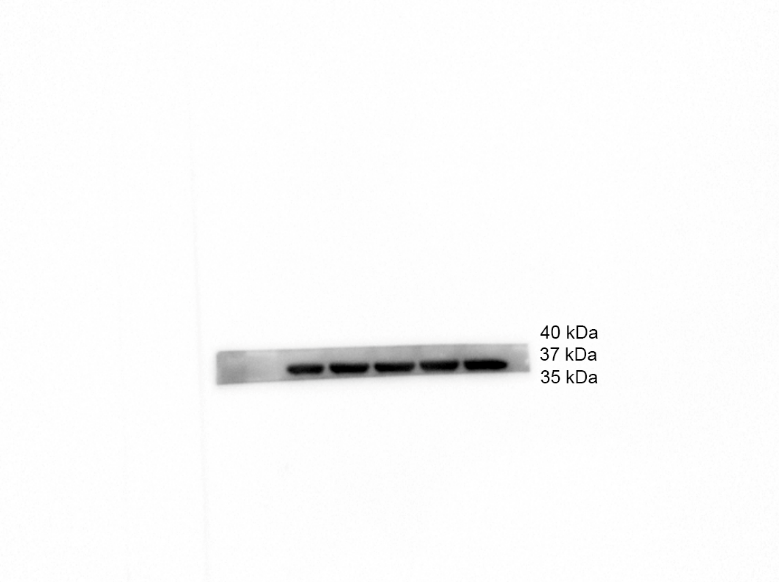


Figure S3G Nrf2 110kDa


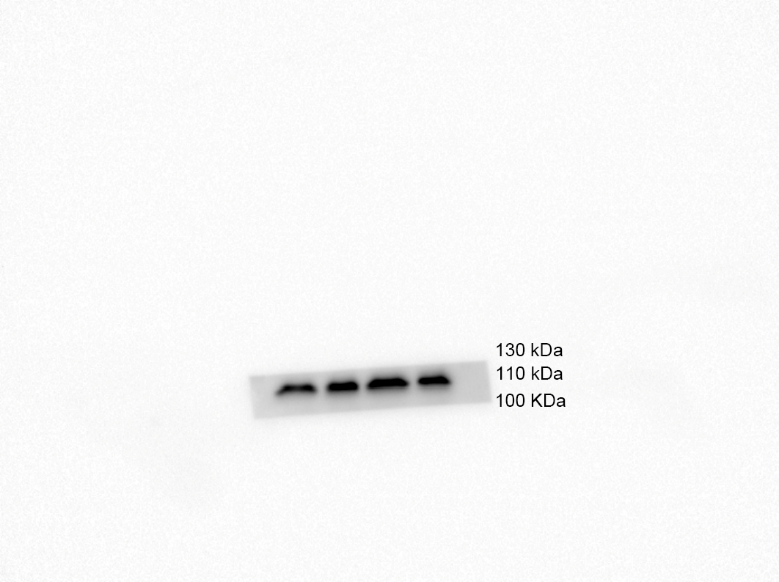


Figure S3G GAPDH 37kDa


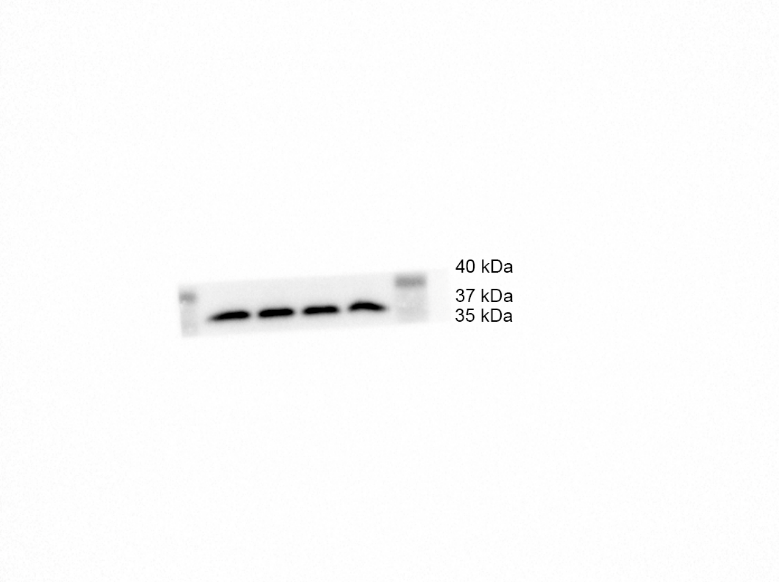

Supplement: Supplementary file 3 — Supplementary material for the original image of WB [file 41419_2022_4592_MOESM3_ESM.docx]
